# miR-17-92 fine-tunes MYC expression and function to ensure optimal B cell lymphoma growth

**DOI:** 10.1038/ncomms9725

**Published:** 2015-11-10

**Authors:** Marija Mihailovich, Michael Bremang, Valeria Spadotto, Daniele Musiani, Elena Vitale, Gabriele Varano, Federico Zambelli, Francesco M. Mancuso, David A. Cairns, Giulio Pavesi, Stefano Casola, Tiziana Bonaldi

**Affiliations:** 1 Department of Experimental Oncology, European Institute of Oncology, Via Adamello 16, Milan 20139, Italy; 2 Units of Genetics of B cells and lymphomas, IFOM, FIRC Institute of Molecular Oncology Foundation, Milan 20139, Italy; 3 Department of Biosciences, Milan University, Milan 20133, Italy

## Abstract

The synergism between c-MYC and miR-17-19b, a truncated version of the miR-17-92 cluster, is well-documented during tumor initiation. However, little is known about miR-17-19b function in established cancers. Here we investigate the role of miR-17-19b in c-MYC-driven lymphomas by integrating SILAC-based quantitative proteomics, transcriptomics and 3′ untranslated region (UTR) analysis upon miR-17-19b overexpression. We identify over one hundred miR-17-19b targets, of which 40% are co-regulated by c-MYC. Downregulation of a new miR-17/20 target, checkpoint kinase 2 (Chek2), increases the recruitment of HuR to *c-MYC* transcripts, resulting in the inhibition of c-MYC translation and thus interfering with *in vivo* tumor growth. Hence, in established lymphomas, miR-17-19b fine-tunes c-MYC activity through a tight control of its function and expression, ultimately ensuring cancer cell homeostasis. Our data highlight the plasticity of miRNA function, reflecting changes in the mRNA landscape and 3′ UTR shortening at different stages of tumorigenesis.

## Abstract (web summary)

The synergism between c-MYC and miR-17-19b plays an important role in lymphoma initiation. In this study, the authors identify a panel of targets co-regulated by miR-17-19b and in *MYC*-driven lymphoma and unravel the molecular mechanism through which miR-17-19b inhibits *MYC* translation.

## Graphical abstract

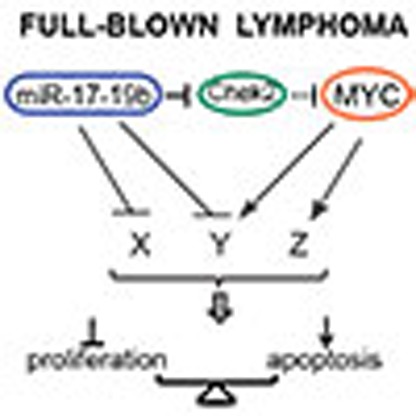

Cellular homeostasis consists in the ability to maintain the internal equilibrium in spite of a changing environment. The intrinsic capability of the cell to maintain homeostasis relies on biological robustness^1^. If this equilibrium is broken, the cell undergoes either uncontrolled proliferation or programmed death.

c-MYC (hereafter referred to as MYC) binds to 10–15% of genomic loci in mammals^2^. MYC governs many critical cellular functions, including energy and anabolic metabolism, proliferation and survival^3^. It promotes on the one hand cell growth and cell cycle progression and, on the other, it sensitizes cells to undergo apoptosis. Thus, under normal circumstances, MYC-induced cell proliferation is counterbalanced by MYC-induced cell death. Deregulation of MYC expression and/or activity is tightly linked to tumour development, as ∼70% of human cancers show aberrant MYC function. MYC expression is regulated at multiple levels, including transcription, translation and protein stability. At the level of translation, MYC is regulated, respectively, by an internal ribosome entry site (IRES) located within the 5′ UTR, RNA-binding proteins including HuR and AUF1, which bind to AU-rich elements located in the 3′ UTR, and various microRNAs (miRNAs)^4^,^5^,^6^. Interestingly, in addition to miRNAs that regulate *MYC* expression, MYC itself regulates the expression of a broad repertoire of miRNAs, many of which are key modulators of cell death and proliferation^7^.

As post-transcriptional silencers of gene expression, miRNAs play a crucial role in increasing robustness of phenotypic outcomes^8^. One way by which miRNAs confer robustness to the cell is through miRNA-mediated feed-forward loops (FFLs), whereby a transcription factor (TF) and a miRNA regulate the same set of protein-coding genes, with the miRNA being regulated by the same TF^9^,^10^. An example of this regulatory circuit is offered by the interplay between the miR-17-92 cluster, the TF E2F1 and MYC^9^. MYC and E2F1 are central regulators of cell cycle progression and apoptosis and thereby play an essential role in cellular homeostasis. Since MYC and E2F1 activate each other at the transcriptional level, there is the risk for the cell to enter a runaway positive feedback loop, resulting in excessively high levels of these transcriptional regulators. However, both factors induce the transcription of miR-17-92, which, in turn, negatively regulates E2F1 translation^11^, thus acting as a break on this positive feedback loop.

miR-17-92 is a polycistron encoding six miRNAs that can be grouped into four families, based on their seed regions: miR-17, miR-18, miR-19 and miR-92. miR-17 and miR-19 families are composed of pairs of miRNAs with identical seed regions: miR-17/miR-20a and miR-19a/miR-19b-1^12^. As oncomirs, these miRNAs promote proliferation, inhibit apoptosis and induce tumour angiogenesis^13^,^14^. Yet, in some contexts, the miR-17 family negatively regulates cell proliferation^15^,^16^,^17^ and inhibits cell migration and invasion^18^,^19^. Therefore, it has become widely accepted that miR-17-92 has the potential to act either as an oncogene or as a tumour suppressor, depending on the cellular context.

Interestingly, in the last few years an increasing body of evidences has shown that 3′ UTRs undergo significant shortening during tumorigenesis^20^. Since 3′ UTR shortening alters the pool of mRNA targets of a given miRNA, this may determine distinct outcomes of the same miRNA's activity at different stages of tumour development.

The interplay between miR-17-92 and MYC has already been extensively studied during MYC-dependent B cell lymphomagenesis. The enforced expression of the truncated version of the cluster, miR-17-19b, was shown to synergize with MYC in accelerating tumorigenesis in the Eμ-MYC mouse lymphoma model^21^. miR-19 was identified as the main effector of this synergism, by counteracting MYC-induced apoptosis through PTEN silencing^22^,^23^. Yet, in spite of the wealth of information collected on the activity of miR-17-19b during lymphoma onset, the role of the cluster in established MYC-dependent tumours remains largely unknown.

In this study, we address the function of miR-17-19b in established MYC lymphomas, at a stage when MYC has pervasively reprogrammed the transcriptome of the tumour cell. By applying an integrated approach centred on SILAC (Stable Isotope Labelling by Amino acids in Cell culture^24^)-based quantitative proteomics, transcriptomics and 3′ UTR analysis, we identify more than a hundred miR-17-19b targets. A large portion of identified targets (about 40%) is predicted to be under the control of MYC, highlighting miRNA-mediated FFLs as an important mode of gene regulation. We also reveal that miR-17-19b indirectly downregulates MYC translation through the newly identified miR-17-19b target, checkpoint kinase 2 (Chek2). Downregulation of Chek2 by miR17/20 leads to increased recruitment of HuR/RISC to *MYC* mRNA, which inhibits its translation. In line with these results, we observe that a subtle increase in miR-17-19b levels reduces the fitness of lymphoma cells, both *in vitro* and *in vivo*.

In conclusion, our data demonstrate that miR-17-19b sustains homeostasis of MYC-driven lymphomas by fine-tuning MYC expression, thus protecting tumour cells from harmful effects linked to excessive MYC levels.

## Results

### Mild perturbation of miR-17-19b affects lymphoma cell proteome

**Figure 1:**
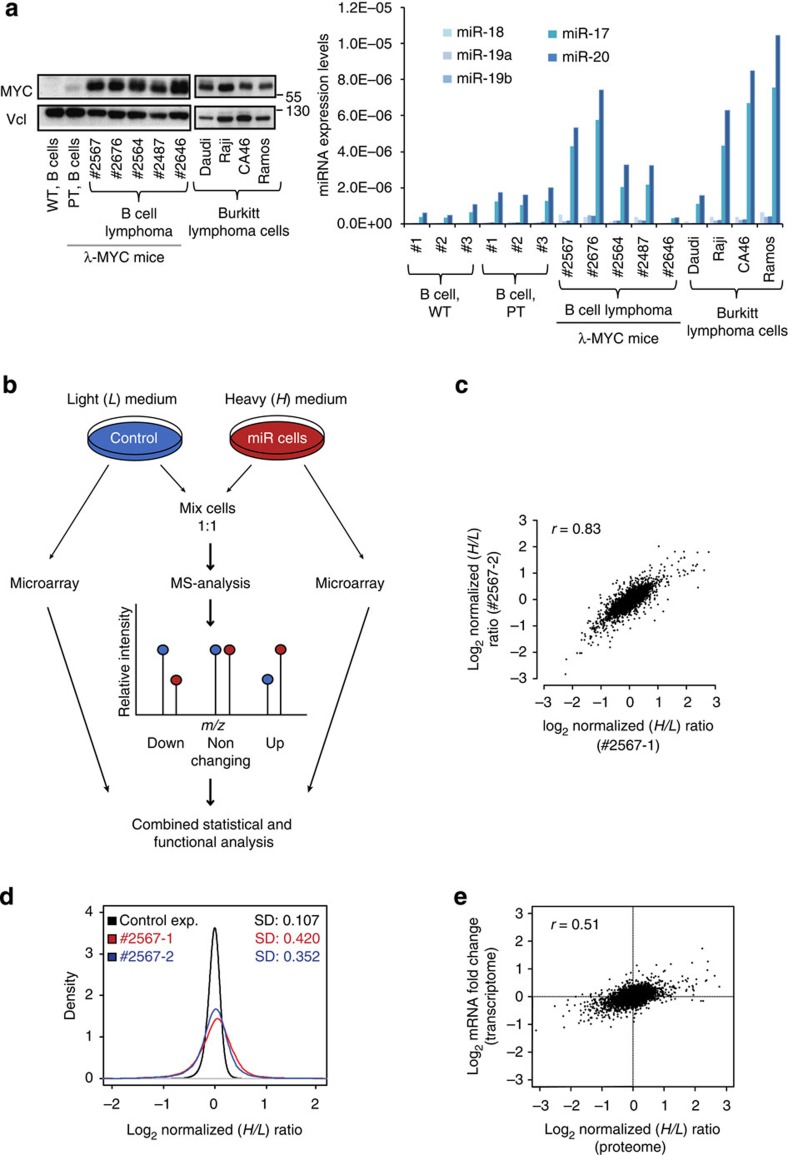
Mild overexpression of miR-17-19b in B cell lymphoma triggers a global proteomic response. (**a**) Expression levels of MYC (left panel) and the mature forms of miR-17-19b components (right panel) were profiled in Epstein–Barr virus (EBV) positive (Daudi and Raji) and EBV negative (CA46 and Ramos) human Burkitt lymphoma cell lines, as well as in primary lymphomas and pre-tumoral (PT) B cells isolated from λ-MYC transgenic mice. Wild-type mouse splenic B cells were used as controls. (**b**) Scheme of the SILAC experiment. Control and miR-17-19b overexpressing cells (miR cells) were cultured in light (L) and heavy (H) media, respectively. Labelled cells were combined in 1:1 ratio and analysed by liquid chromatography–tandem mass spectrometry. In parallel, from the same samples, total RNA was prepared and analysed by microarray. Data were subjected to statistical and functional analysis. Intensity peak ratios between heavy and light peptides (*H*/*L* ratio) reflect changes in protein expression. (**c**) Reproducibility of the SILAC proteome in the two experimental data sets, #2567-1 and #2567-2 (**d**) Log_2_-transformed *H*/*L* protein ratio distributions of the two functional experiments (miR cells versus control for #2567-1 in red and #2567-2 in blue) and of the control experiment (control cells versus control cells, in black) indicate a strong proteome response upon induction of the cluster, with both, up- and downregulated proteins. (**e**) The comparison of log_2_ fold changes at protein and mRNA levels shows low correlation (*r*=0.51), with significantly larger protein dispersion. (See related Supplementary Figs 1 and 2).

We profiled the expression levels of both MYC and the mature forms of miR-17-19b members in human Burkitt lymphoma (BL) cell lines and in *MYC*-transformed pre-tumoral and mature B cell lymphomas isolated from **λ**-MYC transgenic mice^25^. We observed a substantial increase in MYC protein levels at the transition between pre-tumoral and tumoral B cells, which correlated with increased levels of miR-17-19b members in most mouse and human BL cells (Fig. 1a). This result is consistent with previous observations showing MYC-dependent transcriptional activation of the miRNA cluster^11^.

To investigate the role of miR-17-19b in full-blown B cell lymphomas, we perturbed the system by increasing the expression of the cluster in λ-MYC lymphoma B cells, whose endogenous miR-17-19b levels are comparable to those of human BL (Fig. 1a, right panel). We infected lymphoma cells with a retroviral vector encoding miR-17-19b or, as a control, a retrovirus lacking the cluster (Supplementary Fig. 1a). Both vectors expressed green fluorescence protein (GFP) as a reporter, which was used to enrich for infected cells by cell sorting. Global miRNA expression analysis of sorted miRNA-infected cells (miR cells) revealed two- to fivefold higher expression levels of miR-17-19b members, whereas other miRNAs were not affected (Supplementary Fig. 1b). Quantitative reverse transcription–PCR analysis (RT–qPCR) indicated a preferential overexpression of miR-17 and miR-20 (Supplementary Fig. 1c, left panel), showing a relative miRNAs stoichiometry similar to that of the endogenous cluster (Fig. 1a, right panel). The increased level of transgenic miRNAs was also reflected by their engagement within the RISC complex, as monitored by real-time PCR analysis of miRNAs co-immunoprecipitated by Ago2 (Supplementary Fig. 1c, right panel).

To identify miR-17-19b targets we made use of a systems biology approach that integrates *in silico* miRNA-target prediction with quantitative proteomics and transcriptomics analysis on acute induction of the cluster. We employed SILAC-based quantitative proteomics to determine the impact of miRNAs on global protein output. In parallel, a transcriptome analysis was performed using microarray profiling. This approach provided a comprehensive picture of fluctuations in both mRNA and protein levels that resulted from modulation of miR-17-19b expression (Fig. 1b; Supplementary Fig. 2). We identified and quantified over 4,500 proteins with high precision and statistical confidence, and profiled transcript levels of almost 21,000 genes (Supplementary Table 1). High reproducibility of the two replicates (#2567-1 and #2567-2) was confirmed by an overlap of 85% for identified proteins (Supplementary Fig. 2c) and the high correlation (*r*=0.83) between heavy/light (*H*/*L*) protein ratios measured in the two data sets (Fig. 1c).

Gene ontology (GO) analysis of the transcriptome and proteome data showed that although the proteome represents a subset of the transcriptome, it is not biased towards or against any specific functional category (Supplementary Fig. 2d). miRNA-overexpressing tumour cells responded significantly to the modulation of miR-17-19b, showing both up and downregulation of a number of proteins (Fig. 1d). The larger dispersion in protein fold changes compared with transcripts revealed the prevalent response of the proteome to the modulation of miR-17-19b (Fig. 1e). Western blot and real-time PCR analyses confirmed the results obtained by SILAC and microarrays, respectively (Supplementary Fig. 2e,f).

**Figure 2:**
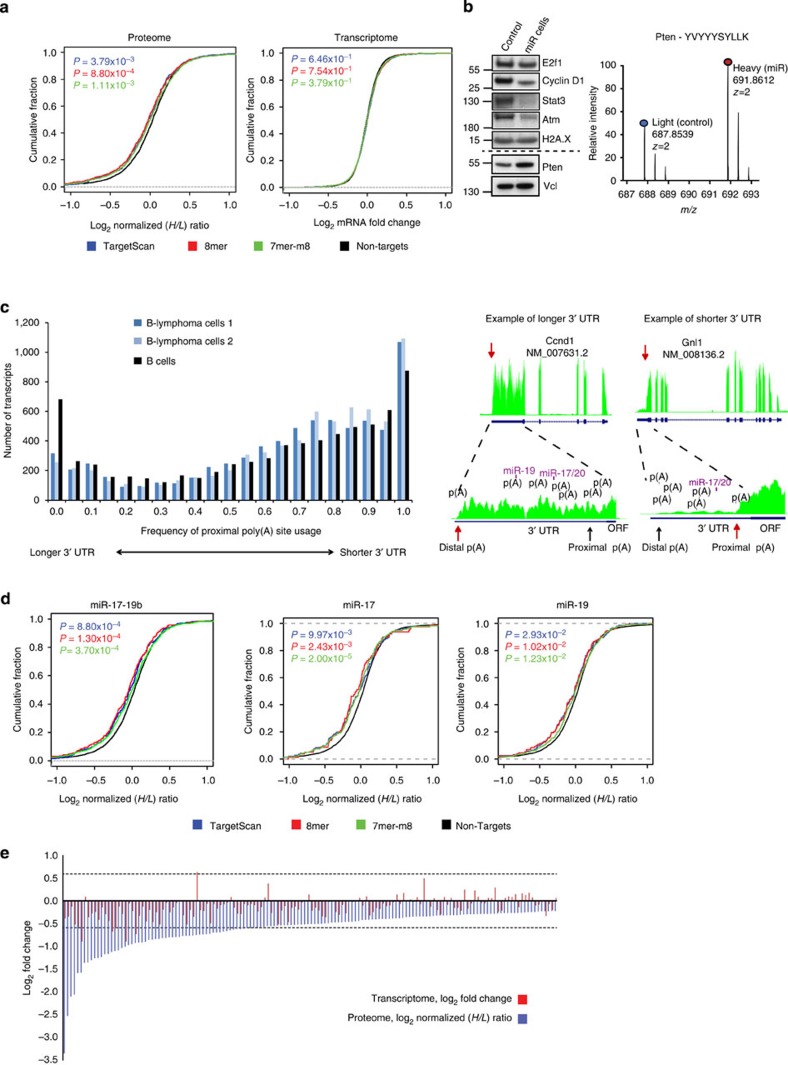
3′ UTR shortening alters the pool of miR-17-19b targets in full-blown lymphoma. (**a**) Cumulative distributions of normalized protein *H*/*L* ratios (left panel) and mRNA fold changes (right panel) upon miR-17-19b overexpression, shown for non-targets (black), miR-17-19b targets predicted by TargetScan (blue), an in-house algorithm for unbiased searching of sites corresponding to 8mer (red) and 7mer-m8 seeds (green). (**b**) Western blot validation of a set of known miR-17-19b targets (left panel; see related Supplementary Fig. 3a). Dotted line separates targets that are confirmed (upper part) from Pten, which is not downregulated in our model (lower part). H2A.X and Vcl were used as loading controls. SILAC-based MS analysis indicates that Pten is not downregulated upon miR-17-19b overexpression (right panel). (**c**) Analysis of proximal poly(A) site usage in two samples from primary λ-MYC B-lymphoma cells (#2567; in blue) reveals a trend towards the usage of shorter 3′ UTRs in a full-blown lymphoma relative to B cells (in black; ENCODE project). The frequency of proximal poly(A) site usage is plotted on the *x*-axis, while number of transcripts is plotted on the *y*-axis (left panel). Examples of distal (*Ccnd1* mRNA) and proximal (*Gnl1* mRNA) poly(A) signal usage, which generate longer and shorter 3′ UTRs, respectively (right panel). Red arrows indicate the poly(A) sites that are used. UTR, untranslated region; ORF, open reading frame; p(A), poly(A). See related Supplementary Fig. 3b. (**d**) Cumulative distributions of normalized protein *H*/*L* ratios for the cluster and for miR-17 and miR-19 families, upon manual filtering for the presence of miR-17-19b seeds within 3′ UTRs. Non-targets are shown in black, miR-17-19b targets predicted by TargetScan are in blue and those predicted by the in-house algorithm for unbiased searching of sites corresponding to 8mer in red and 7mer-m8 seeds in green. (See related Supplementary Fig. 3c). (**e**) Log_2_ fold changes of 148 significantly downregulated miR-17-19b targets at the protein level (blue, for the definition of the statistical cut-off see Supplementary Fig. 4) and mRNA levels (red, #2567-1). The dotted lines represent the cut-off values of ±1.5-fold change that set significant outliers in the transcriptome. (See related Supplementary Table 2).

To identify miR-17-19b direct targets, we intersected the experimental data with a list of genes predicted as targets of the miRNA cluster using two different algorithms. With TargetScan, we identified seed-matching sequences that are also evolutionary conserved. A second algorithm, developed in-house, complemented the first approach and searched in an unbiased manner for 7mer and 8mer seed-matching sequences genome wide. We compared the downregulation of predicted targets to non-targets at both the protein and transcript levels. While we could observe mild but statistically significant responses at the protein level, no significant response was detected at the corresponding mRNA levels (Fig. 2a). We confirmed the downregulation of several known miR-17-92 targets, such as E2f1 (ref. 11), Cyclin D1 (ref. 17), Stat3 (ref. 26) and Atm^27^ upon miR-17-19b overexpression (Fig. 2b and Supplementary Fig. 3a). Surprisingly, instead, tumour suppressor Pten, identified as a miR-17-19b target in the Eμ-Myc lymphoma model^22^,^23^, was not downregulated in established λ-MYC lymphomas (Fig. 2b).

In light of the extensive 3′ UTR shortening observed in cancer cells^20^, we considered the possibility that this phenomenon could affect significantly the repertoire of miR-17-19b targets in established lymphomas. Hence, we performed RNA-Seq analysis of λ-MYC transgenic B-lymphoma cells, focusing in particular on 3′ UTR regions. A comparison of our data with publicly available RNA-Seq data sets obtained from mature B cells of healthy mice indicated a general depletion of transcripts with longer 3′ UTR isoforms and a corresponding enrichment (*P*<2.2e−16, calculated by two-sided Wilcoxon signed rank test) for shorter mRNA isoforms in tumour cells (Fig. 2c, left panel). Interestingly, in tumour B cells, the RNA-Seq analysis identified several *Pten* mRNA isoforms with variable 3′ UTR lengths, including a large proportion of transcripts with sensibly shorter 3′ UTRs, without any miR-17-19b-binding site (Supplementary Fig. 3b). This observation supports the concept that 3′ UTR shortening may contribute to the ‘escape' of *Pten* from miR-19-dependent silencing.

Inspired by this finding, we manually curated all miR-17-19b putative target mRNAs, analysing their 3′ UTRs to verify the actual presence of miR-17-19b-binding sites (Fig. 2c, right panel). With this set of filtered targets, we re-analysed the global miRNA response at the proteome level, to assess the contribution of the cluster and also of individual miRNAs. Indeed, the cumulative distribution analysis showed improved response for the whole cluster and statistically significant downregulation of miR-17 and miR-19 predicted targets compared with non-targets (Fig. 2d). We also observed weak evidence for downregulation of miR-18 targets, whereas no significant response was detected for miR-92, which was our negative control since it was not overexpressed in our experimental system (Supplementary Fig. 3c).

Among the predicted targets, 369 were significantly downregulated at the proteome level before 3′ UTR filtering (Supplementary Table 2, High and Low Confidence Targets; see Supplementary Fig. 4 for cut-off definition), and 271 were left after manual curation of 3′ UTRs for actual miR-17-19b-binding sites (Supplementary Fig. 3d). Of these, 148 proteins were selected as the highest confidence class, based on reproducibility among multiple biological replicates (Supplementary Table 2, High Confidence Targets). From this group, only five were significantly downregulated at both the protein and mRNA level, while the rest were regulated at the protein level only (Fig. 2e). In addition, 15 targets downregulated at the transcript level were not detected in the SILAC proteome (Supplementary Table 2, Transcriptome Targets).

### miR-17-19b impinges on MYC function

**Figure 3:**
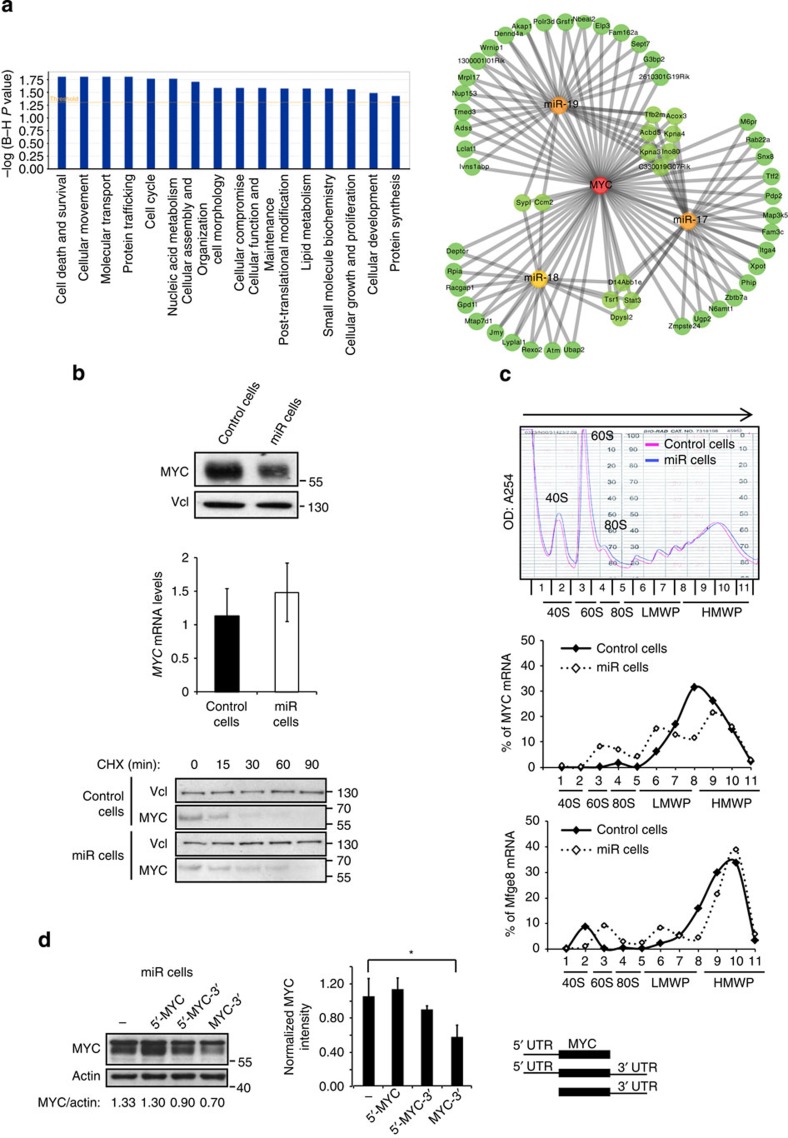
miR-17-19b counterbalances MYC expression and function. (**a**) Ingenuity pathway analysis (IPA) for the newly identified miR-17-19b targets (high and low confidence targets, Supplementary Table 2), left panel. Graphical representation of the MYC-centred regulatory network of the targets co-regulated by MYC and miR-17-19b (right panel). (**b**) Western blot analysis of MYC expression in control and miR cells (upper panel). Real-time PCR analysis of *MYC* mRNA in control and miR cells (middle panel). Histogram represents the averages from three independent experiments with error bars representing s.e.m. MYC expression is normalized to *B2m* mRNA. Western blot analysis of MYC stability in cycloheximide-treated control and miR cells (lower panel; Vcl is used as negative control). (**c**) Polysomal analysis indicates less efficient translation of *MYC* mRNA in miR cells relative to control. Equal amount of the control and miR cells lysates were fractionated through sucrose gradient to generate polysome profiles by measuring absorbance at 254 nm. The relative distribution of *MYC* and *Mfge8* mRNAs (as control) on polysome gradients was assessed by RT–qPCR analysis of the RNA present in all 11 fractions, and displayed as percentage of total mRNA (% of mRNA). Arrow indicates the direction of sedimentation; 40S and 60S, small and large ribosomal subunits, respectively; 80S, monosomes; LMWP (fractions 6–8) and HMWP (fractions 9–11), low- and high-molecular weight polysomes, respectively. The polysome profile from control cells is in pink while that from the miR-17-19b-overexpressing cells is in blue. (**d**) Western blot analysis of MYC protein levels upon enforced expression of constructs bearing a *MYC*-coding region with either a 5′ UTR, 5′ and 3′ UTR or 3′ UTR regions in miR cells (left panel) and the quantification (*n*=3; *t*-test, equal variances, one-tail, *P≤0.05; middle panel) show impaired expression of the construct bearing the 3′ UTR. A schematic representation of the constructs is displayed in the right panel. (See related Supplementary Fig. 5).

Ingenuity pathway analysis (IPA) of the molecular and cellular functions associated with newly identified miR-17-19b targets in λ-MYC lymphomas revealed that several pathways relevant for cancer onset and maintenance are affected, including Cell Death and Cell Cycle Regulation (Fig. 3a, left panel). In addition to known miR-17-92 targets playing key roles in these two processes, such as E2f1 (ref. 11), Cyclin D1 (ref. 17), Stat3 (ref. 26) and Atm^27^, we identified 36 novel miR-17-19b targets involved in their regulation (Supplementary Table 2, Cell Cycle_Apoptosis). Interestingly, several categories, that IPA indicated as modulated by the cluster, are also MYC-regulated processes. Thus, to further explore the cooperation between MYC and miR-17-19b, we intersected the experimental miR-17-19b target data set with a list of MYC targets, inferred from ChIP-Seq data from BL cells^28^. We observed that 40% of the experimentally identified miR-17-19b targets are also bound by MYC in human BL cells (Fig. 3a, right panel). Interestingly, MYC/miRNA-co-regulated targets included several genes encoding TFs, suggesting a model whereby miR-17-19b acts centrally within a multi-layered gene expression network. To further explore this hypothesis, we focused on selected TFs (E2F family members, STAT3 and ZBTB7A), for which predicted targets were available in B cells^29^. The intersection of our experimental target data set with a list of targets for each of the selected TFs confirmed the existence of sub-networks, involving not only these TFs but also their own targets (Supplementary Fig. 5a). Altogether, these data point towards the involvement of miR-17-19b in various gene expression feedback and FFLs, suggesting a role of these miRNAs in buffering a MYC-centred regulatory network against internal and external perturbations.

### miR-17/20 decreases MYC translation via the Chek2/HuR axis

We were prompted to monitor the expression level of MYC itself by the observation that miR-17-19b acts on several MYC-regulated processes. Remarkably, we found that MYC was downregulated upon a mild increase of miR-17-19b, despite not being a direct target of these miRNAs (Fig. 3b, upper panel). To dissect how miR-17-19b regulates MYC expression, we first investigated its transcriptional regulation (Fig. 3b, middle panel) and protein stability by treating cells with cycloheximide, a drug which blocks translational elongation and thus permits monitoring protein stability (Fig. 3b, lower panel). miR-17-19b caused neither significant mRNA downregulation nor reduced MYC protein stability. We then investigated the effect of miR-17-19b on MYC translation. Polysomal analysis revealed increases in sub-monosomes and low molecular weight polysomes, mirrored by a corresponding reduction in heavy molecular weight polysomes of *MYC* transcripts in miR cells compared with control cells (Fig. 3c and Supplementary Fig. 5b). This result indicated that translation of *MYC* mRNA is impaired upon a mild increase of miR-17-19b. By overexpressing distinct *MYC* transgenes that differ in their 5′- and/or 3′ UTRs in miR cells, we could identify the 3′ UTR as the region containing binding sites for putative *trans*-acting factors that negatively regulate *MYC* translation, in a miR-17-19b-dependent manner (Fig. 3d and Supplementary Fig. 5c,d). In the presence of both the 3′- and 5′ UTRs this effect was not observed, suggesting that the 5′ UTR is compensating the effect detected with the 3′ UTR alone. The 5′ UTR of *MYC* contains an IRES which is developmentally controlled^30^. It is active during embryogenesis and downregulated in adult mice. Therefore, it is possible that some factors, required to repress the IRES-regulated translation in adults, are titrated out by excessive levels of the 5′ UTR of MYC, leading to the IRES de-repression.

**Figure 4:**
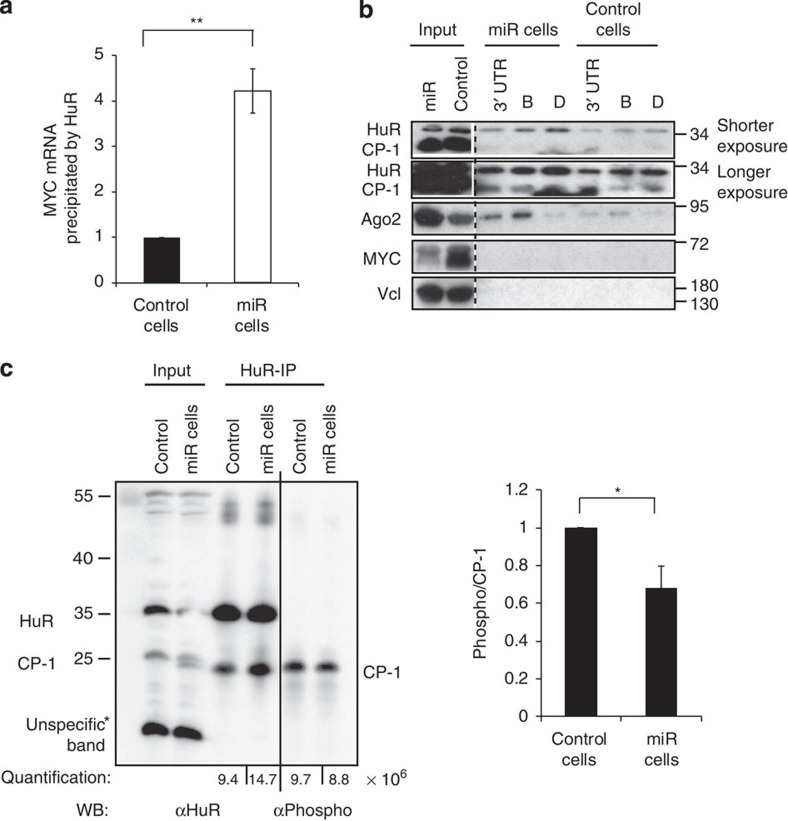
HuR binds more *MYC* mRNA in miR cells. (**a**) HuR-immunoprecipitations (HuR-IPs) coupled with RT–qPCR analysis revealed higher level of *MYC* mRNA precipitated by the HuR in miR cells compared with control. Histogram represents mean±s.e.m. (the control is set as 1; *n*=3; *t*-test, unequal variances, one-tail, **P≤0.01). (**b**) Western blot analysis of HuR, Ago2, MYC and Vcl (negative control) upon RNA pull-down assay. Biotinylated RNAs corresponding to the fragments B and D of *MYC* 3′ UTR (see Supplementary Fig. 6a) were used for pull-down assays with extracts from control and miR cells. Different exposure times were used for input and pull-down results. (**c**) Cytoplasmic extracts from control and miR-17-19b-overexpressing cells were subjected to IPs using anti-HuR antibody (ab). Each HuR-IP was loaded as two samples: 50% IP was probed with anti-HuR and 50% with pS/T ab. The anti-HuR ab precipitated both, the full length (35 kDa) and cleaved form 1 (CP-1, 25 kDa), of which the last is detected as predominantly phosphorylated. A mild, yet reproducible decrease of phospho-HuR was measured in miR cells when compared with control (histogram, right panel). Quantification represents mean of pS/T signal normalized to CP-1±s.e.m. (control cells are set as 1; *n*=3; *t*-test, one-tail, unequal variances, *P≤0.05). See related Supplementary Fig. 6.

It has been previously described that the RNA-binding protein HuR binds *MYC* 3′ UTR and regulates MYC expression in a context-specific way: the full-length protein (35 kDa) can both stimulate^31^ and inhibit^32^ translation, while the cleaved form (25 kDa, cleavage product 1 (CP-1)) inhibits MYC translation^33^. To test the hypothesis that miR-17-19b reduces MYC level by affecting HuR interaction with *MYC* mRNA, we performed ribonucleoprotein-immunoprecipitation (RIP) analysis and we observed that HuR immunoprecipitates higher amounts of *MYC* mRNA in miR cells than in control cells (Fig. 4a). An RNA pull-down assay using as bait four overlapping regions (A–D) of *MYC* 3′ UTR confirmed the preferential binding of HuR to fragments B and D (Supplementary Fig. 6a), in agreement with previous findings^32^. Consistent with the binding of let-7b/c to a region overlapping segments A and B, we observed preferential binding of Ago2 to fragment B (Fig. 4b). Therefore, our results suggest that HuR recruits the Ago2-RISC to finely regulate MYC translation, in accordance with previous data^32^. However, we also detected the CP-1 isoform bound to the same regulatory regions (Fig. 4b, longer exposure), indicating a more complex scenario in our model system than previously reported.

Our SILAC experiment and western blot analysis showed no changes in HuR abundance upon miR-17-19b overexpression (Supplementary Table 1 and Supplementary Fig. 6a, see input in the right panel). Thus, the increased recruitment of HuR to *MYC* 3′ UTR could result from either improved binding or increased translocation of the protein from nucleus to cytoplasm. Since we did not observe any change in the subcellular distribution of HuR (Supplementary Fig. 6b), we focused on the regulation of HuR binding to *MYC* mRNA. The interaction of HuR to a target mRNA is tightly regulated by a complex pattern of phosphorylation at distinct sites, suggesting that a mild increase in miR-17-19b modulates HuR phosphorylation state. To verify this, we compared phospho-HuR in control and miR cells. We performed IP experiments using as baits either anti-HuR or anti-phospho-Serine/Threonine antibodies, followed by western blot analysis using anti-phospho-Serine/Threonine and anti-HuR antibodies, respectively (Fig. 4c left panel, Supplementary Fig. 6c,d). In both experiments, we detected a mild, yet reproducible, decrease of CP-1 phosphorylation in miR cells compared with controls (Fig. 4c, right panel). The phosphorylation on CP-1 was confirmed by treatment with λ protein phosphatase (Supplementary Fig. 6e). Interestingly, phosphorylation seems to occur primarily on CP-1, with the phosphorylation signal on HuR full-length visible only in the anti-Phospho-Serine/Threonine-IP (Supplementary Fig. 6d). This observation hints towards the existence of a correlation between phosphorylation and cleavage of HuR, adding an additional level of complexity to HuR mechanism-of-action.

**Figure 5:**
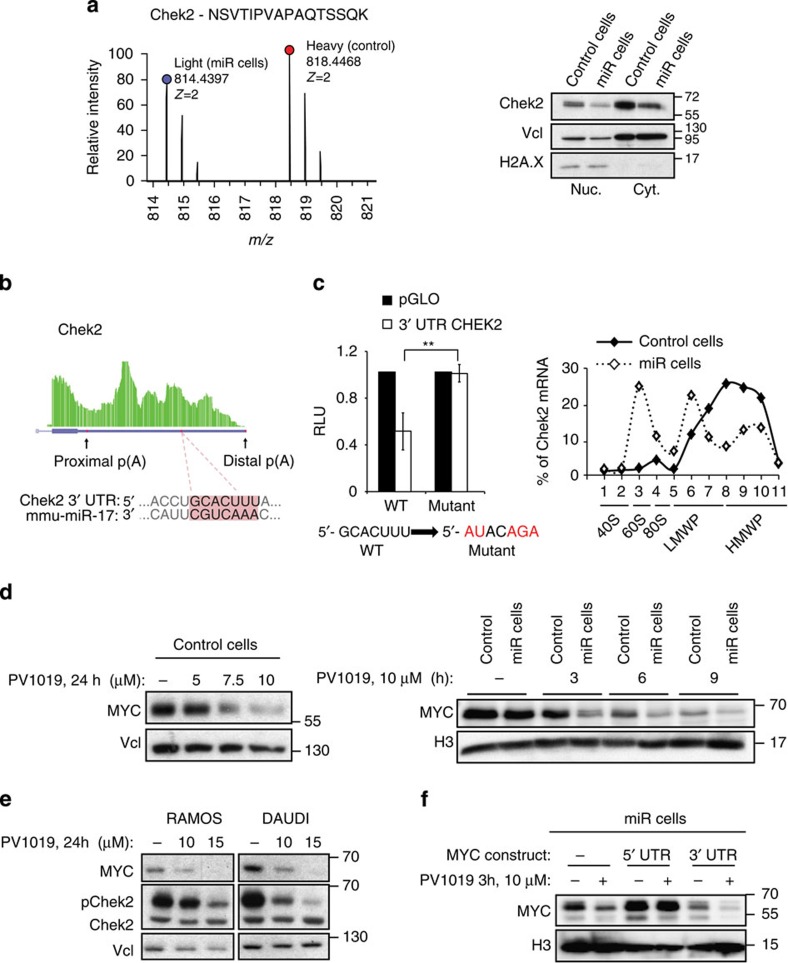
A novel miR-17-19b target Chek2 regulates MYC expression. (**a**) Chek2 is downregulated upon miR-17-19b overexpression, as detected by the SILAC proteomics and western blot analysis (left and right panels, respectively). (**b**) Scheme of Chek2 3′ UTR as obtained from the analysis of RNA-Seq data, with miR-17/20-binding site, proximal and distal p(A) annotated. (**c**) Luciferase reporter assay performed in HeLa cells for a wild-type 3′ UTR Chek2 (WT) or a mutant for the miR-17/20-binding site (left panel). The histogram represents mean±s.e.m. (two biological replicates, done in triplicate; *t*-test, one-tail, equal variances, **P≤0.01). Chek2 mRNA translatability was assessed using the same polysome gradients as for the *MYC* mRNA. Accumulation of Chek2 mRNA in sub-monosomal (40S, 60S and 80S) and LMWP fractions indicate translational repression in the cells overexpressing miR-17-19b (right panel). LMWP and HMWP, low- and high-molecular weight polysomes, respectively. (**d**) Increasing amounts of the Chek2-inhibitor PV1019 progressively downregulate MYC expression in primary B-lymphoma cells. Vinculin (Vcl) was used as a loading control (control cells; left panel). PV1019 time course in control and miR cells revealed stronger response in miR-17-19b-overexpressing cells, where MYC downregulation was detectable already after 3 h; H3 was used as a loading control (right panel). (**e**) PV1019 negatively regulates MYC expression in human BL cells (Ramos and Daudi). Western blot was used to assess levels of total and phosphorylated form of Chek2 (Chek2 and pChek2, respectively). Vinculin was used as a loading control. (**f**) miR-17-19b-dependent regulation of MYC requires Chek2 activity. miR cells, transiently transfected with constructs bearing a *MYC*-coding sequence with either a 5′- or 3′ UTR were treated with PV1019 for 3 h. H3 was used as a loading control.

Although HuR can be phosphorylated by several kinases, such as PKCa, PKCd and Cdk14, Chek2 has been described as the most prominent regulator of HuR binding to target mRNAs^34^. Remarkably, Chek2 emerged as a novel miR-17/20 target from the SILAC experiment and its downregulation was also confirmed by western blot analysis (Supplementary Table 2 and Fig. 5a). *In silico* prediction uncovered a miR-17/20 binding site within the Chek2 3′ UTR, the existence of which was validated by RNA-Seq data (Fig. 5b). The luciferase assay confirmed the downregulation of the reporter gene when fused to the truncated version of the Chek2 3′ UTR bearing the miR-17/20-binding site (Fig. 5c, left panel). Mutation of the miR-17/20-binding site completely rescued the downregulation of the reporter gene (Fig. 5c, left panel). In accordance with these results, endogenous Chek2 transcript accumulated in sub-monosomes and low molecular weight polysomes, indicating translational repression, in cells overexpressing miR-17-19b (Fig. 5c, right panel; and Supplementary Fig. 7a). These data together confirmed Chek2 as a miR-17/20 target in our model.

To corroborate the hypothesis that Chek2 activity affects MYC expression, we used PV1019, a highly selective inhibitor of this kinase^35^. We first validated the effectiveness of the inhibitor in human BL cell lines on the known Chek2 target, Cdc25c (Supplementary Fig. 7b). Twenty-four-hour treatment of λ-MYC lymphoma cells with increasing amounts of PV1019 led to progressive reduction of MYC protein level (Fig. 5d, left panel), without affecting the stability of the protein (Supplementary Fig. 7c). A time course analysis of MYC expression showed that already 3 h after the treatment with 10 μM PV1019, MYC protein levels were significantly reduced, with the effect being more evident in miR cells compared with controls (Fig. 5d, right panel). The same result was observed in the human BL cell lines Ramos and Daudi (Fig. 5e). To pinpoint whether Chek2 activity regulated MYC expression through its 3′ UTR, we employed the constructs bearing either the 5′- or the 3′ UTR region. Upon transient expression of MYC transgenes, PV1019 treatment led to a strong downregulation of the construct bearing the 3′ UTR in miR-17-19b-dependent manner, thus directly linking miR-17-19b regulation of MYC protein level to the kinase activity of Chek2 (Fig. 5f). Moreover, PV1019 treatment reduced HuR phosphorylation (Supplementary Fig. 7d) and boosted its binding to fragments B and D, with consequent decrease of MYC protein level, even after short treatments with the drug (for example, 30 min; Supplementary Fig. 7e).

Taken together, our data suggest that miR-17-19b reduces MYC translational efficiency indirectly, through the downregulation of the novel target Chek2. Chek2 reduction causes increased binding of HuR to *MYC* mRNA, which in turn finely regulates *MYC* mRNA translation.

### MYC/miR-17-19b equilibrium sets B-lymphoma cell fitness

**Figure 6:**
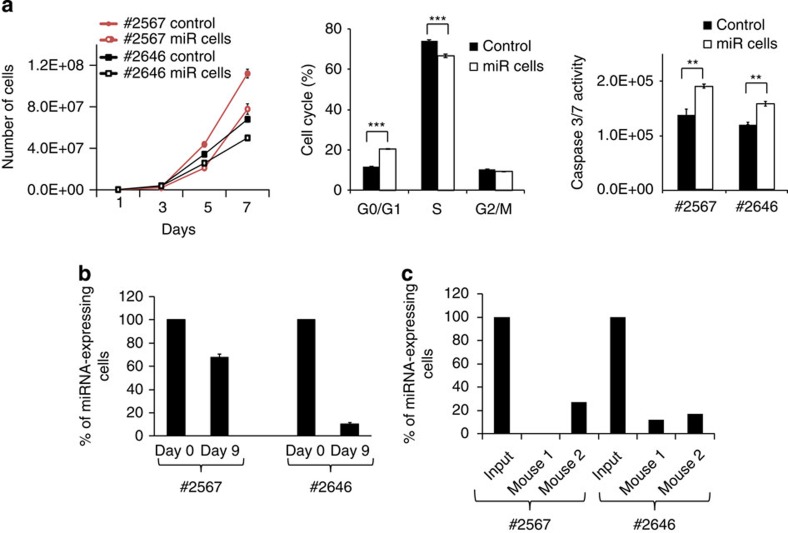
A modest miR-17-19b induction reduces the aggressiveness of MYC-dependent Burkitt lymphoma. (**a**) Phenotypic characterization of miR-17-19b-overexpressing B-lymphoma cells. *In vitro* growth of λ-MYC lymphoma lines (#2567 and #2646) infected with retroviruses expressing miR-17-19b or control virus (left panel). Cell cycle profiles of #2567 cells, assayed by flow cytometry after a short BrdU pulse (middle panel). Apoptosis rate in control and miR cells evaluated by caspase-3/7 activity assay (right panel). Results represent the averages±s.e.m. from three independent experiments (*t*-test, one-tail, equal and unequal variances for cell cycle and apoptosis, respectively, **P≤0.01; ***P≤0.001). (**b**,**c**) miR-17-19b-overexpressing cells are outcompeted by control, when co-cultured both *in vitro* (**b**) and *in vivo* (**c**). Results are represented as % of miR cells relative to the entire population, normalized to the input (see Methods for details of the experiment). For *in vitro* experiments, bar graphs represent the averages±s.e.m. from three independent experiments, while for *in vivo* experiment one sample per animal was analysed in triplicate.

**Figure 7:**
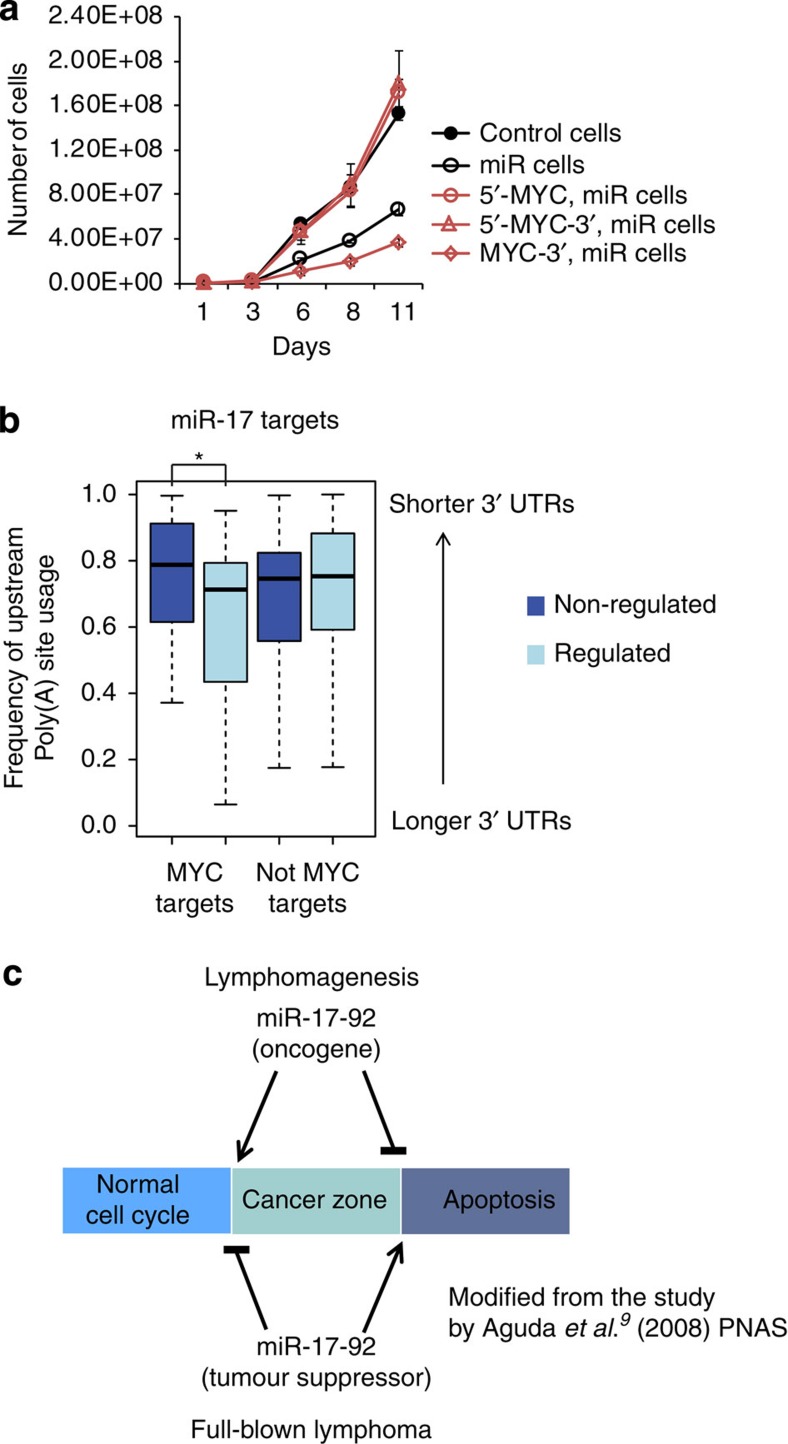
The regulatory loop involving miR-17-92 and MYC maintains lymphoma homeostasis. (**a**) A modest increase of MYC protein level restores growth rate of miR-17-19b-overexpressing cells. Growth rate of the cells is displayed as line plots, which are the mean±s.e.m. from three independent experiments. (**b**) Frequency of poly(A) site usage analysis for ‘regulated' and ‘non-regulated' miR-17 targets. The comparison between regulated and non-regulated targets reveals longer 3′ UTRs for the class of regulated targets, but only when they are co-regulated by MYC. A Mann–Whitney *U* test was used to determine statistical differences between the two distributions (*P≤0.05). (See related Supplementary Fig. 8). (**c**) Proposed model for miR-17-92 role in maintaining lymphoma homeostasis is schematized (modified from the study by Aguda *et al.*^9^). A ‘cancer zone' is defined by a dynamic equilibrium between proliferation and apoptosis. During B cell lymphomagenesis, miR-17-92 antagonizes MYC-induced apoptosis by downregulation of Pten, operating as an oncogene. In full-blown lymphoma, instead, miR-17-92 inhibits cell cycle progression and increases apoptosis through a tight regulation of MYC expression and function, acting as a tumour suppressor.

To assess the biological relevance of miR-17-19b modulation in lymphoma maintenance, we chose two independent λ-MYC lymphomas showing endogenous levels of miR-17-19b similar (#2567) or lower (#2646) than those of human BL (Fig. 1a, right panel). In both λ-MYC lymphomas, a modest increase of miR-17-19b led to impaired proliferation, a reduction in G_1_-to-S transition and a significant increase in the fraction of apoptotic cells (Fig. 6a). The reduction in cell cycle progression, together with impaired tumour survival, resulted in a competitive growth disadvantage of miR cells, which progressively disappeared when co-cultured with their control counterparts (Fig. 6b). A similar effect was also observed when a 1:1 mixture of control and miR transgenic cells was injected into immunoproficient syngeneic mice to give rise to secondary tumours *in vivo*. Real-time PCR performed on genomic DNA isolated from lymphoid organs of tumour-bearing animals revealed the preferential loss of miR cells as revealed by quantification of retroviral DNA specifically carrying the miRNA cluster (Fig. 6c). These results indicate that a mild perturbation of miR-17-19b levels in established MYC lymphomas is sufficient to interfere with *in vivo* tumour growth as a result of miR-17-92 modulation of MYC expression. Indeed, a modest increase in MYC levels (Fig. 3d and Supplementary Fig. 5c) was sufficient to fully restore the fitness of lymphoma cells expressing transgenic miR-17-19b (Fig. 7a).

These results suggest a model whereby MYC activity in tumour cells (including supra-physiological enhancement of anabolic processes) is finely counterbalanced by selected miRNAs to ultimately sustain immortal growth of the tumour. According to this model, MYC may sustain miR-17-92 inhibitory activity, for a subset of co-regulated targets, by selecting mechanisms that prevent 3′ UTRs from shortening. Remarkably, in agreement with this model, we observed that miR-17-19b targets, co-regulated by MYC, contain 3′ UTRs longer than those from miRNA-targets that are not co-regulated by MYC (Fig. 7b and Supplementary Fig. 8). This provides evidence for an essential role of MYC/miR-17-92 FFLs throughout tumour development.

## Discussion

Gene expression silencing mediated by miRNAs can have different effects, depending on the cellular context. Global downregulation of miRNA expression is a common feature of human tumours^36^. Yet, the miR-17-92 cluster is highly expressed in a variety of cancers^21^, suggesting an important role in tumour maintenance. Downregulation of the tumour suppressor Pten by miR-19 was identified as a relevant contribution of the cluster to MYC-driven lymphomagenesis in the pre/pro-B Eμ-MYC lymphoma model^22^,^23^. Remarkably, Pten was not downregulated in established λ-MYC lymphomas upon acute perturbation of miR-17-19b levels. Absence of Pten downregulation may depend on Pten 3′ UTR shortening, which leads to its escape from miR-19 regulation.

The effect of miRNAs on cell function depends on the particular transcriptional profile of the cell. Alternative polyadenylation has only recently gained attention as a key regulator of the dynamics of gene regulation^37^. Any change in the lengths of 3′ UTRs may influence the fate of the mRNA as a result of altered binding of RNA-binding proteins and miRNAs^38^,^39^. For example, in rapidly proliferating T cells, proliferation-related genes, which contain binding sites for miR-155 and miR-17-92, have significantly shorter 3′ UTRs relative to non-targets^40^. Similarly, cancer cells express mRNAs with overall shorter 3′ UTRs, where the shortening was linked to the activation of oncogenes, due to escape from miRNA silencing^20^.

In this study, we made use of several complementary approaches to investigate the effects of miR-17-19b on different levels of gene regulation in malignant B cells. *In silico* prediction analyses of miRNA targets suffers from a high rate of false positives, due to the incomplete sequence complementarity between miRNAs and their targets and the large heterogeneity of 3′ UTRs, as revealed by next generation sequencing technology. The combined strategy described here underlines the power of intersecting quantitative proteomics with in-depth 3′ UTR analysis to gain an accurate and comprehensive picture of miRNA-mediated gene regulation.

Upon acute increase of miR-17-19b expression in MYC-transformed B cells, we observed a predominant response at the proteome level, relative to the transcriptome. These results are consistent with the view that miRNAs control gene expression through translational repression and mRNA degradation^41^,^42^.

Surprisingly, our analysis showed that MYC itself is downregulated upon acute induction of miR-17-19b in lymphoma cells. MYC downregulation has been previously linked to the activity of the miR-17 family in MCF-7 cells^17^. However, the molecular details of this mechanism remained elusive, given that MYC is not a direct target of this miRNA family. Our data suggest that miR-17/20, through downregulation of the Chek2 kinase and the consequent reduction of HuR phosphorylation, increases HuR binding to *MYC* mRNA, leading to inhibition of MYC translation.

The RNA-binding protein HuR/Elav1 (embryonic lethal abnormal vision-like protein 1) plays an important function in mRNA biology (mRNA processing, trafficking, decay and translation). HuR elicits distinct gene expression outcomes in different contexts, including tumorigenesis and/or tumour maintenance^43^,^44^. An additional layer of complexity in HuR biology derives from its ability to modulate translation in a context-specific manner^34^. HuR activity and binding to target mRNAs is regulated by a complex, cell-specific, pattern of post-translational modifications^45^. For example, increasing cellular polyamines in rat intestinal epithelial cells stimulates Chek2 activity and Chek2-dependent HuR S100 phosphorylation, which positively regulates HuR binding to *MYC* mRNA and MYC translation^31^. Of note, mutations of the other two residues targeted by Chek2 cause opposite outcomes. Specifically, while mutation of S88 increases association of HuR to MYC mRNA, mutation of T118 does not impinge on HuR binding. Moreover, the cleaved form of HuR (CP-1) was also shown to repress *MYC* translation on hypoxic stress^33^. Therefore, understanding of how context-specific dynamic changes of HuR post-translational modification patterns modulate HuR activity remains an unachieved, yet challenging goal.

In addition, given the high number of miR-17-19b targets in our system, we cannot exclude that other mechanisms also contribute to the observed downregulation of MYC.

The importance of the fine equilibrium between MYC and miR-17-92 during B cell tumorigenesis has been emphasized by Olive *et al.*^46^, who reported that miR-92, by inhibiting degradation of MYC, stimulates uncontrolled cell growth while increasing at the same time programmed cell death. The authors propose a scenario whereby two distinct miRNAs of the miR-17-92 exert antagonizing functions on the regulation by MYC on apoptosis during lymphomagenesis. Specifically, while miR-92 stimulates p53-dependent apoptosis and ensures elimination of pre-malignant MYC-overexpressing cells, miR-19 represses MYC-induced programmed cell death^46^.

Our study extends this model by adding players and further emphasizes the role of miR-17-92 in guaranteeing the correct balance between opposite biological outcomes in cancer cells (Fig. 7c). The fine-tuning of MYC exerted by miR-17-92 in cancer cells guarantees at any given time point a tight control over its transforming activity, with the ultimate goal to sustain tumour maintenance (Fig. 7c). The acute perturbation of miR-17-19b levels in aggressive lymphomas allowed us to appreciate the fine equilibrium between miR-17-92 and MYC in tumour cells and the biological consequences of this tight balance on *in vivo* tumour growth.

In conclusion, our study illustrates the plasticity of miR-17-19b function, which is tightly connected to dynamic changes in the mRNA landscape that accompany tumour formation and maintenance. At the molecular level, we unravelled the inhibitory effect of miR-17-19b on MYC expression and function in established MYC-driven lymphomas. From a cancer-biology perspective, we envisage a scenario whereby miR-17-19b maintains cellular homeostasis by protecting cancer cells from exceedingly high and potentially harmful MYC levels, ultimately guaranteeing its ability to sustain continuous tumour growth.

## Methods

### Tumour cell lines and cell culture

Mouse B-lymphoma cell lines (#2567, #2646, #2676, #2564 and #2487) were established after *in vitro* culture of primary lymphomas isolated from λ-MYC transgenic mice^25^. Tumours were cultured *in vitro* in B cell medium (DMEM (Invitrogen) supplemented with 10% foetal calf serum (FCS), 100 U ml^−1^ of penicillin and streptomycin (Lonza), 2 mM Glutamine (Lonza), 1 mM non-essential amino acids (Gibco), 1 mM Na-pyruvate (Gibco) and 50 mM β-mercaptoethanol (Gibco)). After 2–3 weeks of *in vitro* culture, an expanding population of IgM^+^B220^+^ lymphoma cells was established. Clonal status of the tumour was confirmed by Southern blotting analysis of Igκ V gene rearrangements. Procedures involving animals were approved by IFOM animal Ethics Committee and the Italian Ministry of Health. Human BL cell lines (CA46, RAMOS, Raji and Daudi) were cultured in RPMI medium (Invitrogen), supplemented with 10% FCS, 100 U ml^−1^ of penicillin and streptomycin, 2 mM Glutamine, 1 mM Na-pyruvate and 1 mM Hepes. All cells were cultured at 37 °C in a humid atmosphere with 5% CO_2_. CA46 and RAMOS cell lines are not present in the ICLAC list of misidentified cell lines, while Daudi and Raji are classified as misidentified. However, both cell lines, Daudi and Raji, are identified as human BL cells, which was the only criteria for the choice of the cell lines used in the study.

### Cloning and retroviral gene transduction

Plasmid pMIG-miR-17-19b was generated by PCR amplification of miR-17-19b, a truncated version of miR-17-92, from mouse genomic DNA (BamHI_miR-17, FW_5′-CGGGATCCGTCAGAATAATGTCAAAGTGCT-3′ XhoI_miR-19b, RV_5′-CCGCTCGAGCACTACCACAGTCAGTTTTGCAT-3′). The amplified fragment was cloned into the GFP containing retrovirus-mediated gene expression vector pMIG II by BamHI–XhoI digestion and DNA sequenced for verification. The virus was produced by Phoenix cells and transfected by Ca_2_Cl protocol in B-lymphoma cell lines. A spin infection was performed in triplicate with a total number of 2 × 10^5^ cells per well. At 48 h post infection, the efficiency of transduction was evaluated by flow cytometric analysis monitoring GFP expression, and subsequently GFP-sorted. All experiments were performed within first 10, post infection, cell passages.

### RNA extraction and real-time PCR analysis

Total RNA was prepared using mirVANA miRNA Isolation kit (Ambion), according to the manufacturer's specification for total RNA isolation. The complementary DNA was produced using the Reverse Transcriptase ImPromII (Promega), starting from 1 μg of total RNA. One-tenth of the reaction was used for real-time PCR reactions with FAST SYBR Green Master Mix (Applied Biosystems) and in 7,900HT Fast Real-Time PCR System. All experiments were performed in triplicate, unless specified. Relative changes in mRNA levels of target genes were calculated by normalizing the amount of transcripts to the levels of *H2a.x* or *B2m*.

For RNA-Seq, total RNA was purified using Trizol (Invitrogen), treated with Turbo DNase (Ambion), purified with phenol-chloroform and precipitated with EtOH. Libraries for RNA-Seq were then prepared with TruSeq RNA Sample Kits v2 (Illumina), following manufacturer's instruction for enrichment of poly(A) mRNAs. RNA-Seq was performed on Illumina HiSeq 2,000 sequencer.

miScript Reverse Transcription Kit, miScript Primer Assay and miScript SYBR Green PCR Kit (Qiagen) were used according to the manufacturer's instructions for miRNAs profiling.

### SILAC experiment

Control and miRNA-expressing cells were grown in SILAC media^24^ (lysine- and arginine-free DMEM/Ham's F12 (1:1), supplemented with 10% dialyzed foetal bovine serum (Invitrogen)). ‘Heavy' and ‘Light' media were prepared by adding 0.146 g l^−1 13^C_6_, ^15^N_2_ L-Lysine and 0.84 g l^−1 13^C_6_^15^N_4_ L-Arginine (Sigma) or the corresponding non-labelled amino acids, respectively. After eight replications, equal numbers (12 × 10^6^) of heavy and light cell populations were mixed and lysed in 300 μl RIPA buffer. Denaturation of protein extracts (150 μg) was achieved by incubation in 6 M urea/2 M thiourea for 30 min, followed by reduction with 1 mM DTT (30 min). Thyols were carboxymethylated with 5 mM IAA for 20 min and the proteins were digested by addition of 1 μg LysC for 3 h. After fourfold dilution with 50 mM ammonium bi-carbonate, 1 μg trypsin was added and the sample was incubated overnight at 37 °C. The digestion was terminated by acidifying the sample to pH<2 with 100% TFA. Product peptides were separated according to their isoelectric point by isoelectrofocusing electrophoresis using the Agilent 3,100 OFFGEL Fractionation Kit (Agilent Technologies)^47^. Samples were prepared and separated according to the manufacturer's protocol. After separation, peptides mixtures were reconstituted with 1% TFA and desalted on C18 STAGE tips^48^.

### Liquid chromatography and Mass spectrometry

Peptides eluted from C18 Stage-tips were analysed by nano-liquid chromatography (LC) using an Agilent 1,100 Series nano-flow LC system (Agilent Technologies), directly interfaced to a LTQ-FT Ultra mass spectrometer (ThermoFisher Scientific, Bremen, Germany). The nanolitre flow LC was operated in one column set-up with a 15 cm analytical column (75 μm inner diameter, 350 μm outer diameter) packed with C18 resin (ReproSil, Pur C18AQ 3 micro-meter, Dr Maisch, Germany). Solvent A was 0.1% FA and 5% ACN in ddH2O and solvent B was 95% ACN with 0.1% FA. Samples were injected in an aqueous 0.1% TFA solution at a flow rate of 500 nl min^−1^. We used 140 min gradient from 2 to 60% acetonitrile in 0.5% acetic acid. Nanoelectrospray ion source (Proxeon, Odense, Denmark) was used with a spray voltage of 1.5–2.0 kV. No sheath, sweep and auxiliary gasses were used and capillary temperature was set to 180 °C. The mass spectrometer was operated in a data-dependent mode to automatically switch between mass spectrometry (MS) and tandem MS (MS/MS) acquisition. In the LTQ-FT Ultra, full scan MS spectra were acquired at a target value of 2,000,000 ions and with a resolution of 100,000 (full width at half maximum) at 400 *m/z*. In the LTQ MS/MS, spectra were acquired using a target value of 5,000 ions and the 5 most intense ions were isolated for CID-fragmentation.

### Analysis of MS data

Identification and quantification of proteins was carried out using the MaxQuant software package v. 1.2.2.5 (refs 49, 50). The integrated Andromeda algorithm was used to search the IPI Mouse protein sequence database (v.3.87, Sep 2011). A maximum of two missed cleavages were permitted. Oxidation of methionine and acetylation of the protein N terminus were used as variable modifications. Carbamidomethylation of cysteine was used as a fixed modification. A minimum peptide length of six residues was required and the maximum false discovery rates were set to 1% at both the protein and peptide levels. MaxQuant-defined protein groups, which matched reversed protein sequences or contaminants, were excluded. To determine at high confidence a qualitative proteome, each protein group required a minimum of two peptide matches, of which at least one was unique from all peptides identified in #2567-1 and #2567-2 combined.

These two data sets were analysed separately for quantification. Therefore, to determine an accurate quantitative proteome, a minimum of three ratio counts (RC>2) was required from peptides identified either in #2567-1 or in #2567-2. Details of the composition of the acquired and filtered proteome are presented in Supplementary Table 1.

### Defining cut-off for definition of responders in the SILAC experiment

To define a cut-off for responders, we made use of a triplicate SILAC proteome of WT:WT cells to examine technical variation in the experimental proteome and define a cut-off point. By examining the protein ratio distribution over the full intensity range and the protein ratios separated into bins of intensities of size 300 (Supplementary Fig. 4a, panels 2–11), and estimating the quantiles at 0.5 and 99.5% (denoted by red horizontal lines also given numerically in Supplementary Fig. 4b) provides limits which describe the range in which 99% of the data lie. The cut-off point used to define responders was taken as the median of the cut-off points for each of the binned proteomes, resulting in the figures in the final row of the table in Supplementary Fig. 4b. This gives at least a 5% false positive rate for classes C–J, 10% in class B and a maximum of 20% in class A.

### GO analysis

The reference genome-based proteome was constructed using all mouse protein identifiers present in the IPI database (v.3.87, September 2011). MGI identifiers corresponding to all genes were mapped to biological process GO terms, using the gene association file made available by the Mouse Genome Informatics database^51^. These GO terms were then matched to GO slim terms, made available by the same database.

### Microarray hybridization and RT–qPCR validation

Affymetrix expression array was performed by Affymetrix-GeneChip Mouse gene 1.0 ST Array hybridization with total RNA from SILAC experiment in three biological replicates. Complementary DNA was synthesized using random hexamers, oligo d(T) and the SuperScript II Reverse transcriptase System (Invitrogen). Real-time PCR validation of microarray data was done by Cogentech (IFOM-IEO Campus), with TaqMan Gene Expression Assays from Applied Biosystems for following genes: Itga4 (Assay ID: mm00439770_m1), Atm (mm01177457_m1), Runx1 (mm00486762_m1), Bcl2l11 (mm01333921_m1), Bax (mm00432050_m1), Ctgf (mm00515790_g1), Pten (mm00477210_m1), E2f3 (mm01138831_m1), Uhrf2 (mm00520043_m1), Cd38 (mm00483146_m1), Ptk2b (mm00552827_m1), Pbk (mm00517793_m1), Pfkp (mm03053257_s1), Slc1a5 (mm00436603_m1), Acin1 (mm00479895_m1), Rasa3 (mm00436272_m1), Gmfb (mm00517681_m1), Aifm1 (mm00442540_m1), Htra2 (mm00444846_g1), Kras (mm00517494_m1), N-ras (mm03053787_s1), Cdc42bpb (mm00805507_m1), Capn (mm00482964_m1), Stat3 (mm03053490_s1), Dffb (mm00432822_m1), Plcg (mm01242530_m1), B2m (mm00437762_m1).

Primers used for mRNA profiling of known miR-17-19b targets, upon miR-17-19b overexpression (*H2ax* was used for normalization):

E2f1_FW: 5′-ATGGGCCACCTGAGGGTCCC-3′;

E2f1_RV: 5′-CCTCGTGGGGTGGGGAGAGG-3′

CyclinD1_FW: 5′-GCTGCAGTGCTGTAGGCCCC-3′;

CyclinD1_RV: 5′-GGCCCTCAAGGGTCCTGCCT-3′

Stat3_FW: 5′-GGGCGACCCTATCGTGCAGC-3′;

Stat3_RV: 5′-GACTAAGGGCCGGTCCGGGT-3′

Atm_FW: 5′-TCTGCTGTCTGCGCACGTCC-3′;

Atm_RV: 5′-GCAGGAGTTGCTGAGCGGCT-3′

H2ax_FW: 5′-CGTGGTCTCTCAGCGTTGTTCGC-3′;

H2ax_RV: 5′-TGAAGGCCGGCGCGTGAAGA-3′.

### Analysis of microarray data

Expression profiling of total RNAs was performed on raw.CEL files using the R statistical programming language (R Development Core Team, Vienna) and tools from the Bioconductor project^52^ and the aroma.affymetrix package^53^,^54^. The robust multi-chip average method was used for background adjustment, normalization and summarization^55^ and quality control information was also made available. Annotation data were extracted using the mogene10sttranscriptcluster.db R/Bioconductor package.

### Cell extracts and western blot analysis

Total cell extracts were prepared in standard RIPA buffer (10 mM Tris-HCl, pH 8.0, 1% Triton, 0.1% SDS, 0.1% Deoxycholate, 140 mM NaCl, 1 mM EDTA, 1 mM DTT, 1 mM PMSF and protease inhibitor cocktail (Roche, Basel, Switzerland)). Nuclear and cytoplasmic fractions were prepared as described previously^56^. Briefly, the cytoplasmic fraction was obtained by lysing cells in a low salt buffer (100 mM Hepes, pH 6.8, 5 mM KCl, 5 mM MgCl_2_, 0.5% NP-40, 5 mg l^−1^ Aprotonin, 5 mg l^−1^ Leupeptin, 0.5 mM PMSF, 0.5 mM Na-butyrate, 5 mM Na-vanadate and 5 mM NaF), while for the nuclear extract in a high salt buffer containing 250 mM NaCl. DNA was digested with Benzonase for 10 min at 37 °C. Western blots were performed following standard protocols: protein extracts were resolved by SDS–PAGE, transferred to PVDF membrane (Immobilon-P, Millipore) using Bio-rad electrophoresis systems and probed with primary and secondary antibodies in 5% milk solved in TBS with 0.1% Tween. Proteins were visualized by using the ECL system (Amersham or Bio-Rad). Primary antibodies against MYC (sc-N-262), Ppp2r2a (PP2A B55-alpha 2G9; sc-81606), E2F1 (KH95; sc-251), GMF-β (C-17; sc-46999), Cdkn1b (sc-528) and N-RAS (C-20 sc-519) were purchased from Santa Cruz; Stat 3 (9132), Atm (2873), Pten (138G6), Aifm1 (AIF, 4642), Bax (2772), PBK/T0PK (4942), Ptk2b (PYK2; 3292), Ccnd1 (DC56) and Ube2a (5293) from Cell Signaling; H2AX (ab11175), Htra2 (ab75982) and PFKP (ab72680) from AbCam; Vcl (V9131) from Sigma and CHK2 from Millipore. Vcl antibody was used at dilution 1:20,000, while all other antibodies were used at 1:1,000 dilution.

Chemiluminescent signal was detected by a film or by ChemiDoc XRS+ (Bio-Rad), which was also used for the quantification of the chemiluminescent signal. The full blots are shown in Supplementary Figure 9.

### Seed analysis to identify miRNA targets

The seed analysis was performed considering four miRNA-families, miR-17 (comprise miR-17 and miR-20), miR-18, miR-19 (comprise miR-19a and miR-19b) and miR-92. Two approaches were used to detect potential miR-17-19b-binding sites for all protein-coding mouse genes. First, conserved target sites were identified using TargetScan Mouse v6.2 (ref. 57). Then, 7mer-m8 and 8mer sites were identified also in an unbiased manner using a custom Perl script. This script was applied to all isoforms of mouse 3′ UTR sequences, obtained from Ensembl v67 (ref. 58) using the Biomart data-mining tool^59^.

### RNA-Seq data and poly(A) sites usage analysis

RNA-Seq data sets of about 200–250 million paired end reads each were aligned to the mm9 mouse reference genome using Tophat2 in conjunction with Bowtie, with default parameters^60^,^61^,^62^. More than 85% of the sequences were mapped. Reads failing to map both on mouse transcriptome or genome were mapped on the locus of the human *MYC* gene, to assess the transcriptional activity of the transgene, resulting in ∼500,000 more reads mapped per sample (0.2%).

The mouse poly(A)-Seq annotations of ‘Merck Research Laboratories' from the UCSC Genome Browser database^63^ reports strand-specific poly(A) sites found in five mouse tissues (brain, kidney, liver, testis and muscle). We combined these data in a unique strand-specific putative poly(A) site list, merging adjacent sites when their distance was <20 bp. We assigned the resulting poly(A) sites to the corresponding mouse RefSeq mRNAs and non-coding RNAs. For each of the 7,157 RNAs with at least 2 putative poly(A) sites and an average coverage greater than 10 in at least one of our samples, we computed the average read coverage in a 100-bp window upstream of the first (most upstream, proximal) and the last (most downstream, distal) poly(A) site of the RNA. We employed as control adult (8 weeks) B cell (CD19+) RNA-Seq coverage data, from the ENCODE project, available at the UCSC Genome Browser database. Differential poly(A) site usage was estimated as follows: *U=(A*_f_*—A*_l_)/*A*_f_, where *A*_f_ and *A*_l_ are the average read coverage in the 100-bp windows before the first and last poly(A) site, respectively. Values of *U* close to 0 thus indicated preference for poly(A) sites at the 3′ of the transcript, while values close to 1 pointed towards preferential usage of upstream poly(A) sites.

### Statistical analysis for frequency of proximal poly(A) site usage

For all high confidence miRNA targets, each family of miRNA targets was separated into two groups of targets: those that were joint MYC targets and those with no evidence for MYC regulation. The distributions of proximal poly(A) site usage (see ‘RNA-Seq data and poly(A) sites usage analysis') were displayed in box plots and compared through a Mann–Whitney *U*-test, where *P*<0.05 implies a significant difference between the two distributions.

### Selection of miR-17-19b target genes

The list of computationally identified mouse genes containing one or more potential miR-17-19b-binding sites was intersected with quantified proteome and transcriptome data. Targets of different miRNAs were considered to be significantly downregulated if their associated protein expression was measured in either #2567-1 or #2567-2 with >2 ratio counts and a normalized *H*/*L* ratio <0.855. This cut-off was derived through an assessment of technical variation in the experimental proteome. miRNA targets were considered to be downregulated at the transcriptome level if the fold change was log_2_<−0.5. We inspected manually the expression of 3′ UTR regions in our RNA-Seq data to ensure that the miRNA-binding sites were present in expressed isoforms. Genome coordinates (mm9) of all predicted miRNA-binding sites were determined and uploaded to the UCSC genome browser, and were displayed along with RNA-Seq data from Sample 1. A total of 1,478 miRNA targets, robustly quantified in our proteomics data, was inspected manually and annotated as either: YES, YES*, NO or ND. YES implies strong evidence for the presence of at least one binding site for any miRNAs from the cluster, while YES* refers to weak evidence. NO implies strong evidence that the expressed 3′ UTR does not contain any miRNA-binding site for miRNAs from the cluster. No Data implies that the coordinates determined for the miR-17-19b-binding sites were outside the annotated boundaries of all transcripts related to the gene framework, which could derive from differences between Ensembl and RefSeq databases.

### Cumulative distributions to display regulatory effect of miRNA action

Normalized *H*/*L* protein ratio distributions for miRNA-target and non-target genes were compared for each seed type: Targetscan, unbiased 8mer and unbiased 7mer-m8. The significance of expression downregulation between these groups was evaluated using a one-sided Kolmogorov–Smirnov test. This downregulation was visualized using empirical cumulative distribution function (ECDF) curves. To discriminate the effect of individual miRNAs, this analysis was performed both for combined and separate members of the miR-17-92 cluster. A *P* value <0.05 inferred significant downregulation of miRNA targets relative to non-targets. Similar analysis was performed for transcript fold changes. All statistical analyses were performed in R/Bioconductor software.

### Construction of MYC/miRNA FFLs

We intersected the list of MYC gene targets obtained from the study by Seitz *et al.*^28^, (accession: GSE30726) with the experimentally derived miR-17-19b responders (high and low confidence targets, Supplementary Table 2). Transcriptional targets of E2F1, E2F3, E2F4, E2F5, LRF and STAT3 were obtained from protein–DNA interactions annotated in predicted B cell interactome data^29^. Lists of targets for each TF were intersected with the list of miR-17-19b targets. Statistical significance of enrichment of jointly regulated targets was evaluated by Fisher's exact test (*P*<0.05, two-sided).

### HuR and Ago2 RIP

HuR-IP was performed as previously described^64^. To prepare cytosolic extracts, control and miR cells were lysed with passive lysis buffer (20 mM Hepes pH 7.4, 100 mM KCl, 0.3% NP-40, 5 mM MgCl_2_, protease inhibitors (Roche), 1 mM Na_3_VO_4_, 50 mM NaF and 10 mM β glycerol-phosphate). Extracts were pre-cleared with mouse IgGs (sc-2025) for 30 min, at 4 °C. IgGs were removed by incubating extracts with protein-G magnetic beads (Dynabeads, Invitrogen) for 30 min, at 4 °C for. Pre-clearing was performed in IP buffer 150 mM NaCl, 100 mM Tris-HCl pH 7.4, 1 mM DTT, 1 mM EDTA. Extracts were incubated for 2 h at 4 °C with beads previously coupled with HuR antibody (Abcam, ab136542) overnight. Beads were washed four times with IP buffer. Bound proteins were eluted with Laemmli buffer, and analysed by SDS–PAGE coupled with immunoblotting. Ago2-IP was performed starting from 2 × 10^6^ cells by using the RiboCluster Profiler RIP-Assay Kit (MBL), according to the manufacturer's instructions with anti-mouse Ago2 antibody (018–22021, Wako).

### RNA–biotin pull-down analysis

RNA pull-down assay was performed as previously described^64^ with following modifications: primers for the transcripts spanning the 150-nt fragments from 3′ UTR of *MYC* mRNA (NM_002467) were from Kim *et al.*^32^ The T7 RNA polymerase sequence

T7: 5′-CCAAGCTTCTAATACGACTCACTATAGGGAGA-3′.

3′ UTR-A (1891–2042):

FW_5′-(T7)GGAAAAGTAAGGAAAACGATTCC-3′;

RV_5′-CCAATTTGAGGCAGTTTACATTATG-3′;

3′ UTR-B (1991–2146):

FW_5′-(T7)GCTGAGTCTTGAGACTGAAAGA-3′;

RV_5′-CATTGTGTAAATCTTAAAATTTTT-3′;

3′ UTR-C (2096–2250):

FW_5′-(T7)CAGATTTGTATTTAAGAATTGTT-3′;

RV_5′-TACCTATAATACTAGGTACTAT-3′;

3′ UTR-D (2199–2357):

FW_5′-(T7)GCAGTTACACAGAATTTCAATCCT-3′;

RV_5′-TTTTTTTTTTAAGATTTGGCTCAATGA-3′.

### Polysome analysis

Polysome analyses from B cells were done as previously described^65^. Briefly, 40 ml of 7.5 × 10^5^ cells per ml in exponential phase were treated with 0.1 mg ml^−1^ cycloheximide for 10 min at 37 °C, washed twice with ice-cold PBS and lysed in 350 μl of polysome extraction buffer (PEB: 15 mM Tris pH 7.4, 15 mM MgCl_2_, 0.3 M NaCl, 15% Triton-100, 0.1 mg ml^−1^ cycloheximide, 40 U ml^−1^ RNasin, complete protease inhibitor cocktail). Extracts were incubated 10 min on ice and centrifuged at 13,000 r.p.m. for 10 min at +4 °C. Supernatant (equal amount of material or supernatant from equal starting number of cells) was layered onto 10 ml 10–50% sucrose gradient (prepared in PEB without Triton-100) and ultracentrifuged at 35,000 r.p.m. for 190 min in a SW41 rotor at +4 °C. Fractions of 1 ml were collected. Prior to the RNA extraction, 1 ng of luciferase mRNA synthesized *in vitro* was added as internal control for the extraction of the RNA. Polysomal fractions were treated with 10 μl 10% SDS, 25 μl 0.5 M EDTA and 4 μl 20 mg ml^−1^ proteinase K for 30 min at 55 °C. RNA was isolated with Quick-RNA Mini prep (ZYMO RESEARCH), and afterwards analysed by RT–qPCR. Obtained qPCR values for specific genes were normalized to luciferase.

### Luciferase reporter assay

Primers corresponding to 62 nt of Chek2 3′ UTR containing miR-17/20-binding site wild-type (3′ UTR WT) or mutated (3′ UTR MUT)^66^ were annealed and cloned downstream of the luciferase reporter gene into the pmirGLO vector (Promega) using XbaI and SacI restriction enzymes. HeLa cells were seeded in 48-well dishes at 4 × 10^4^ cells per ml. The day after cells were transfected with 0.5 μg of pmirGLO, pmirGLO—3′UTR WT and pmirGLO—3′UTR MUT using Lipofectamine LTX (Invitrogen). About 24 h after transfection, luciferase signal was assessed using Dual-Glo Luciferase Assay System (Promega) according to the manufacturer's instructions.

(3′ UTR WT: FW-5′-CCGTGGAAATCTGGGCTGATTGTTTGATGACCTGCACTTTAGATTTCTTTCTTTCTTTCTTTCT-3′;

RV-5′-CTAGAGAAAGAAAGAAAGAAAGAAATCTAAAGTGCAGGTCATCAAACAATCAGCCCAGATTTCCACGGAGCT-3′;

3′ UTR MUT: FW-5′-CCGTGGAAATCTGGGCTGATTGTTTGATGACCTGATACAGAGATTTCTTTCTTTCTTTCTTTCT-3′;

RV-5′-CTAGAGAAAGAAAGAAAGAAAGAAATCTCTGTATCAGGTCATCAAACAATCAGCCCAGATTTCCACGGAGCT-3′).

### Ingenuity pathway analysis

IPA was done with standard settings (mammals—all tissues and cell lines; experimentally observed and high predicted confidence).

### Electroporation

Plasmids overexpressing *MYC*-coding region with either a 5′ UTR, 5′ and 3′ UTR or 3′ UTR^67^ were electroporated by using Microporator Digital Bio according to manufacturer's instruction. Briefly, 2 μg of DNA and 1.3 × 10^6^ cells were resuspended in 100 μl buffer T and pulsed with 990 V for a duration of 40 ms. Electroporated cells were seeded in multiwall 12 (two electroporations per well) in the final volume of 1 ml of medium. The next day, cells were brought to the concentration of 0.1 × 10^6^ cells in the presence of 0.5 μg ml^−1^ of puromycin.

### Preparation of B cell suspension from lymphoid organs

Single-cell suspensions were obtained by grinding spleens and lymph nodes between frosted slide glasses followed by filtering through 70 μm nylon meshes (Becton Dickinson, USA). Femurs were flushed using a syringe containing 10 ml of B cell medium to obtain bone marrow cells. Erythrocyte lysis from spleen was achieved by incubating cell suspensions in 1 ml of a 9:1 (v/v) solution of 0.15 M NH_4_Cl and 0.17 M Tris-HCl, pH 7.65 for 3 min on ice. Red blood cell lysis was interrupted adding 10 vol of B cell medium. Cells were centrifuged at 1,200 r.p.m. at 4 °C and resuspended in B cell medium before counting.

B cells were purified from a heterogeneous cell suspension by magnetic cell sorting (MACS; Miltenyi Biotec, Germany). Cell populations were stained with anti-CD19-antibodies conjugated with magnetic beads (10 μl per 10^6^ cells) for 30 min on ice and washed twice with degased MACS buffer (PBS, pH 7.2, supplemented with 0.5% bovine serum albumin, 2 mM EDTA; degas buffer by applying vacuum). Next, cells were applied to LS columns in a magnetic field^68^ and the columns were washed three times with 3 ml of MACS buffer. Columns were then removed from the magnetic field, 5 ml of B cell medium were added and CD19-positive cells were flushed out using a plunger. Purity achieved by MACS was over 95%. Purified cells were washed once with cold PBS and pelleted for RNA and protein extraction.

### Growth curve, cell cycle profiling and apoptosis assay

Growth curves were generated by seeding 2 × 10^5^ cells per well in 12-well plates, in triplicate. At 2-day intervals, cells were counted and the average number of cells was calculated. The experiment was done in three biological replicates.

For assessment of proliferative status of #2567-1 cells, control and miRNA-expressing cells were pulsed with bromodeoxyuridine (BrdU, Sigma) for 5 min. After extensive washings, cells were fixed and prepared for FACS analysis using standard procedure. Briefly, 2 × 10^6^ cells were fixed in 70% ethanol for 30 min at 4 °C, denatured in 2 N HCl for 25 min at room temperature, neutralized with 0.1 M sodium borate for 2 min and stained with anti-BrdU antibody (Beckton Dickinson) for 1 h at room temperature in 1% BSA/PBS. Anti-mouse Alexa 647, diluted in 1% BSA/PBS for 1 h at room temperature was used as the secondary antibody. Cells were finally resuspended in 1 ml PI (0.025 mg ml^−1^) containing 0.25 mg ml^−1^ RNase A and incubated overnight at 4 °C. FACS data were collected using a FACSCalibur (BD), and data analysis performed with CellQuest and Flowjo software. For apoptosis assay, the Apo-ONE Homogeneous Caspase-3/7 Assay (Promega) was used according to the manufacturer's instructions. Briefly, 1 × 10^5^ cells in exponential phase were mixed 1:1 with provided caspase-3/7 substrate. Caspase activity was measured after 18 h of incubation at room temperature with constant agitation. The experiment was done in three biological replicates, each with three technical replicates.

### Competition assay

For *in vitro* competition assay, control and miRNA cells were seeded in 1:1 ratio (total number of cells 2 × 10^5^ cells per well, seeded at 1 × 10^5^ cells per ml). Genomic DNA was extracted at indicated time points by using DNeasy Blood & Tissue Kit (Qiagen). The relative proportion of the two populations was estimated by real-time PCR profiling of genomic DNA, measuring total GFP amount and exogenous miRNAs. The specific measurement of the exogenous miRNAs was assured by designing set of primers of which one lays within the vector and the other is within the miR-17-92 cluster (B2m_FW: 5′-GGCTCACACTGAATTCACCCCCAC-3′; B2m_RV: 5′-ACATGTCTCGATCCCAGTAGACGGT-3′; miR_FW: 5′-CCATCTACTGCATTACGAGCA-3′; miR_RV: 5′-CGCACACCGGCCTTATTC-3′; GFP_FW: 5′-TGCAGTGCTTCGCCCGCTACC-3′; GFP_RV: 5′-CCGTCGCCGATGGGGGTGTTC-3′).

For *in vivo* competition, control and miRNA cells were mixed in equal amount (1:1) and washed twice in PBS. Cells were resuspended in PBS at 3 × 10^7^ cells per ml and 100 μl of cell suspension was injected into the tail vein of 16–20-week-old immunoproficient CB6F1 mice. From the same 1:1 mixture, 1 × 10^6^ cells were stored before transplantation as input sample. Lymphocytes were collected from the spleen of sick animals and subsequently the relative proportion of the two populations was estimated by real-time PCR. Obtained data were normalized to *B2M* and input samples. Results are represented as % of miRNA-expressed cells versus whole population.

## Additional information

**Accession codes:** The mass spectrometry proteomics data have been deposited to the ProteomeXchange Consortium^69^ via the PRIDE partner repository, with the accession code PXD002810.

**How to cite this article:** Mihailovich, M. *et al.* miR-17-92 fine-tunes MYC expression and function to ensure optimal B cell lymphoma growth. *Nat. Commun.* 6:8725 doi: 10.1038/ncomms9725 (2015).

## Supplementary Material

Supplementary Information Supplementary Figures 1-9
