# Supplementary material for: miR-17-92 fine-tunes MYC expression and function to ensure optimal B cell lymphoma growth
Source: Nat Commun. 2015 Nov 10;6:8725. doi: 10.1038/ncomms9725 (PMC4667639; doi:10.1038/ncomms9725)

Supplementary Information

Mihailovich M. et al., 2015 Supplementary Fig 1

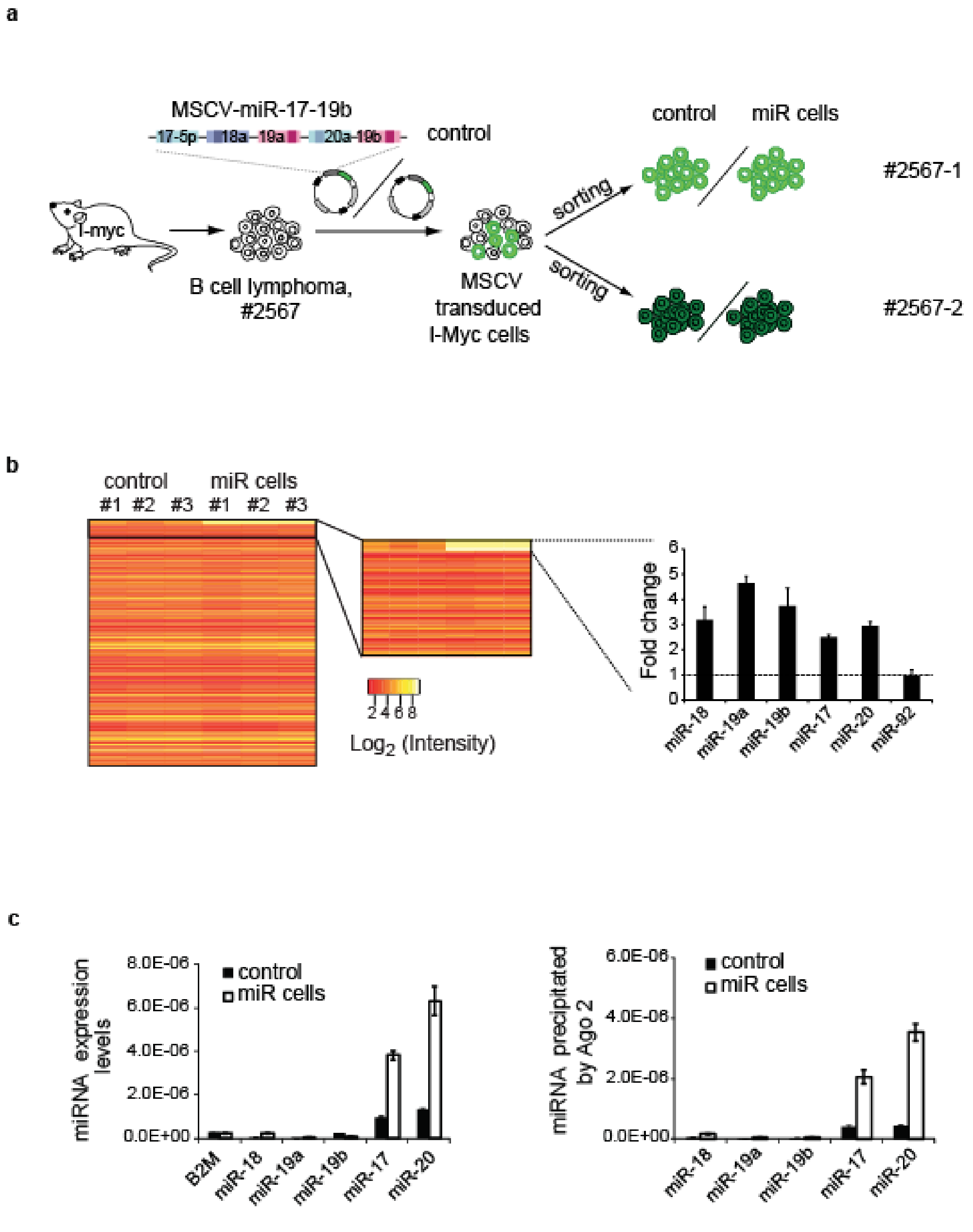

Supplementary Figure 1, related to Figure 1. Enforced expression of miR-17-19b does not affect expression of the endogenous miRNAs. (a) Schematic representation for the generation of primary lymphoma B cell lines overexpressing miR-17-19b. **(b)** Microarray analysis of 367 miRNAs upon miR-17-19b overexpression in the λ-MYC tumor cells (clone #2567) for three experimental samples is shown. The mean ± SEM (Standard Error of the Mean) of three microarray experiments, for the mature forms of miR-17-92 members, is represented as a bar graph. **(c)** Profiling of the mature miRNAs of the cluster in total RNA (left panel) and upon Ago2-IP (right panel) in control and miR-17-19b overexpressing cells. Bar graphs represent the averages ± SEM from three biological replicates for total RNA profiling, and for three technical replicates for Ago2-IP.

Mihailovich M. *et al.*, 2015 Supplementary Fig 2

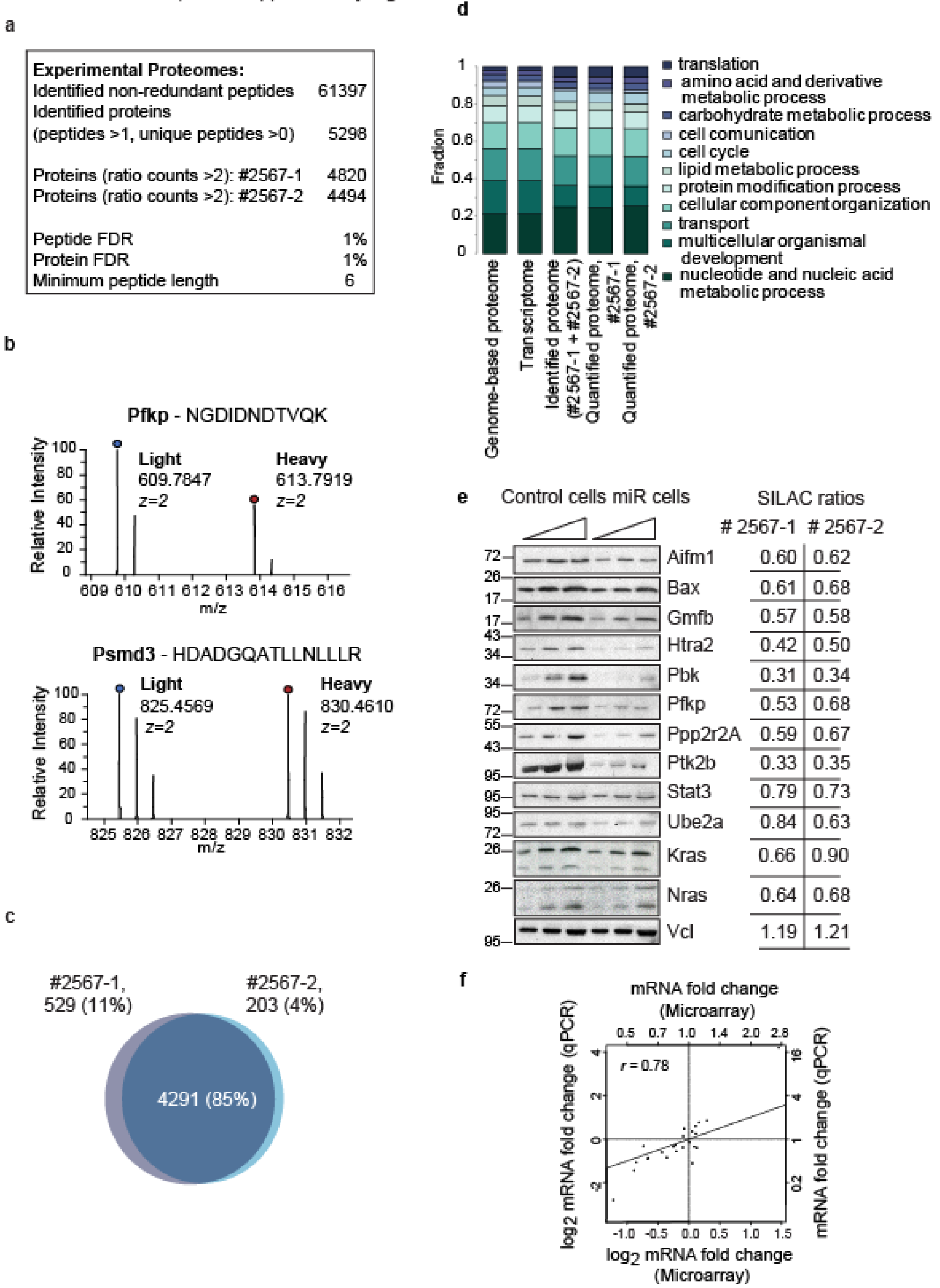

Supplementary Figure 2, related to Figure 1. Validation of the acquired quantitative proteome and transcriptome. **(a)** Features of the acquired SILAC-based proteomes. **(b)** Examples of peptide mass spectra with SILAC pairs for the two functional states (light = control; heavy= miR cells), for a down-regulated and non-changing protein upon miR-17-19b overexpression. **(c)** Venn diagram of quantified proteins in #2567-1 and #2567-2 cells. **(d)** Gene Ontology analysis of biological processes for acquired proteomes and transcriptome. **(e)** Western blot validation of SILAC experiments with indicated normalized H/L ratios obtained in #2567-1 and #2567-2 cells. Vcl was used as loading control. **(f)** Real-time PCR validation of microarray data.

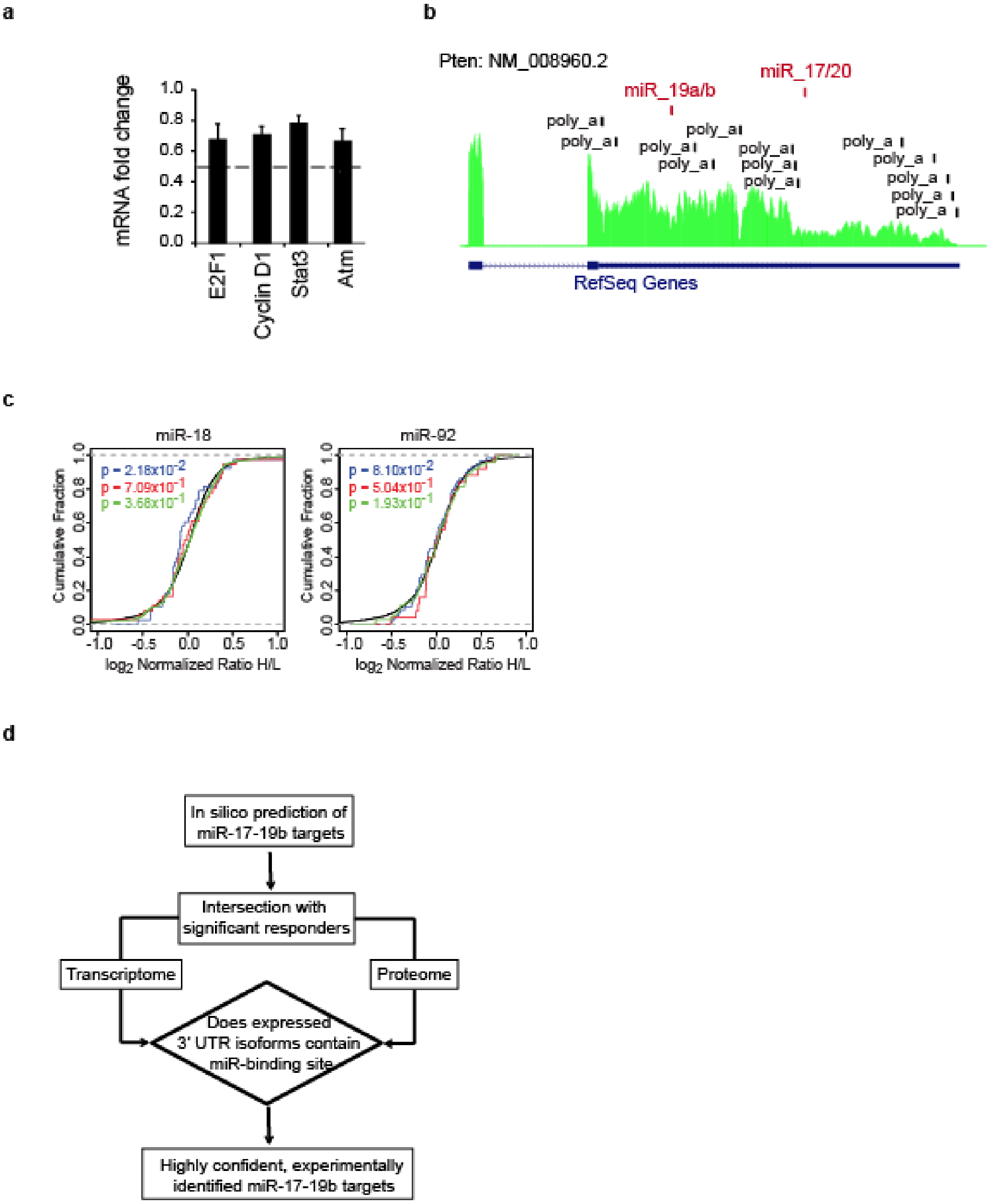

Supplementary Figure 3, related to Figure 2. Pten undergoes alternative polyadenylation in full-blown lymphoma. (a) Real-time PCR validation of some known miR-17-19b targets. Dotted line corresponds to 1.5 fold change cut-off. Values are normalized to the mRNA level of H2A.X. Bar graph represents the averages ± SEM from three independent experiments. **(b)** RNA-Seq analysis uncovered a mixed population of *Pten* mRNA isoforms, with 3' UTRs of different lengths. **(c)** The cumulative distributions of normalized protein H/L ratios for miR-18 and miR-92 families, shown for non-targets (black) and miR-17-19b targets predicted by: TargetScan (blue), an in-house algorithm for unbiased searching of sites corresponding to 8mer (red) and 7mer-m8 seeds (green). Only the targets manually inspected for the presence of miR-17-92 seeds within 3' UTRs were used. **(d)** Data analysis pipeline for target identification.

a

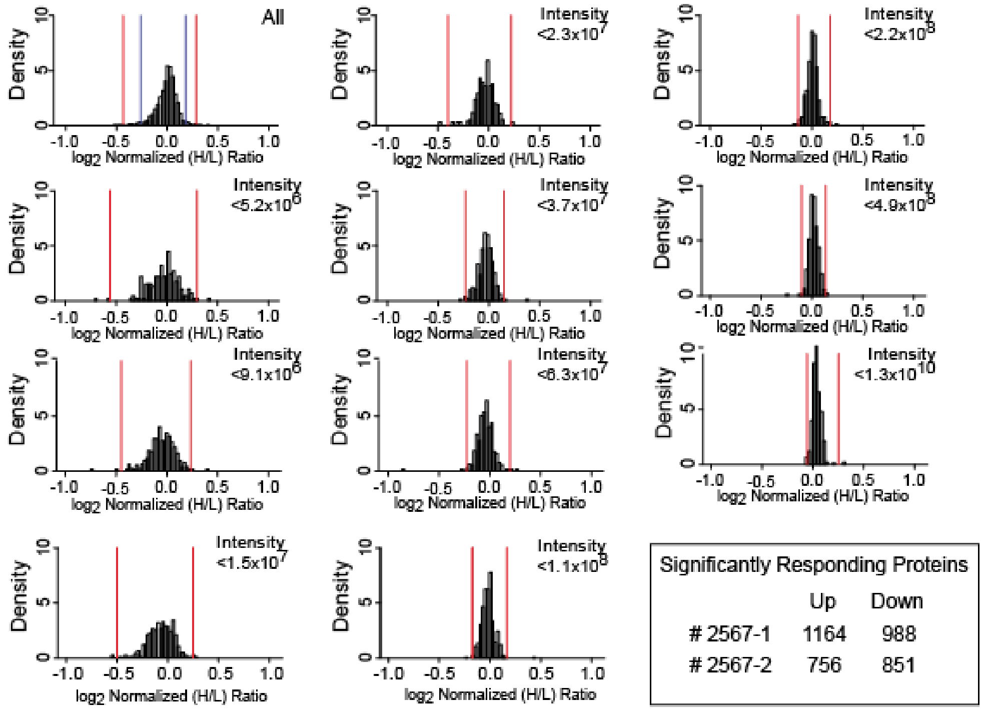

Significantly Responding Proteins	Up	Down
# 2567-1	1164	988
# 2567-2	756	851

b

Intensity Group	Minimum Intensity	Maximum Intensity	Low Responder Cut-off Point	High Responder Cut-off Point
A	0	5185000	0.659	1.242
B	5185000	9114000	0.723	1.165
C	9114000	14740000	0.711	1.197
D	14740000	23340000	0.748	1.169
E	23340000	37430000	0.848	1.112
F	37430000	62890000	0.862	1.165
G	62890000	111000000	0.891	1.128
H	111000000	223800000	0.901	1.124
I	223800000	493600000	0.924	1.090
J	493600000	13160000000	0.959	1.196
Median			0.855	1.165

Supplementary Figure 4, related to Figure 2. Cut-off definition. (a) Protein ratio distributions from control cells, obtained by combining three technical replicates. The first panel shows the distribution for all proteins and the remaining show protein ratio distributions for bins of 300 in increasing protein intensity. The red horizontal lines indicate the 0.5% and 99.5% quantiles in each distribution, clearly demonstrating the large spread of ratios in low abundant proteins with small intensities and the reduced spread as abundance increases. The blue horizontal lines in the first panel represent the 2.5% and 97.5% quantiles. The number of significantly up- and down-regulated proteins in #2567-1 and #2567-2 are indicated. **(b)** The exact intensity values used to bin the data by intensity and numerical values for 0.5% and 99.5% quantiles, displayed by red horizontal lines. The median of the upper and lower cut-off points are presented in the final row and are used to define responders.

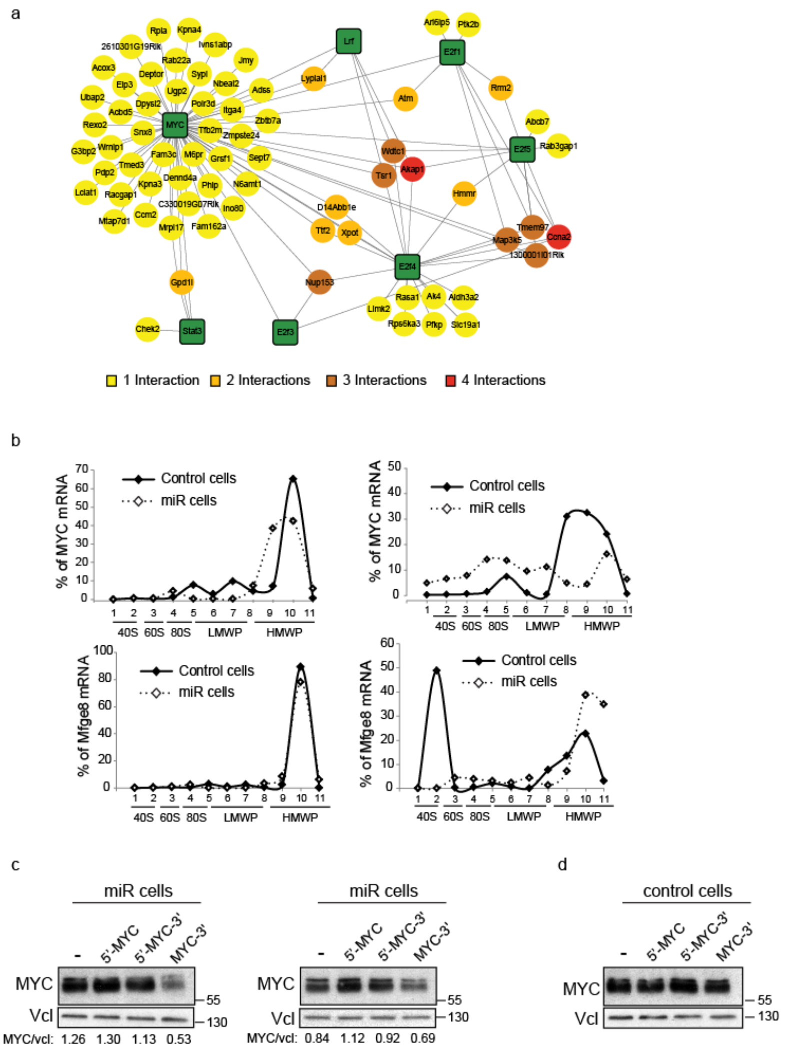

Supplementary Figure 5, related to Figure 3. Multi-layered network centered on MYC-miR-17-92. **(a)** Significantly down-regulated targets of miR-17-19b that are co-regulated by MYC, STAT3, LRF and/or by the E2F family members are displayed. Simple circuits involving one transcription factor are displayed in yellow. Targets regulated by 2, 3 or 4 transcription factors are shown in orange, brown and red, respectively. **(b)** Polysomal analysis indicates less efficient translation of *MYC* mRNA in miRNA-overexpressing cells relative to control. Equal amounts of cytoplasmic lysates (upper panel) or equal starting number of control and miR cells (lower panel) were fractionated through a sucrose gradient to generate polysome profiles. The relative distribution of *MYC* and *Mfge8* mRNAs (as control) along the polysome gradients was assessed by RT-qPCR analysis of the mRNAs present in the 11 fractions and plotted as percentage of total mRNA (% of mRNA). 40S and 60S = small and large ribosomal subunits, respectively; 80S = monosomes; LMWP (fractions 6-8) and HMWP (fractions 9-11) = low- and high molecular weight polysomes, respectively. **(c, d)** Western blot analysis of MYC protein levels upon enforced expression of constructs bearing a *MYC*-coding region with either a 5' UTR, 5' and 3' UTR or 3' UTR regions in miR cells (panel c) and control (panel d).

Mihailovich M. et al., 2015 Supplementary Fig 6

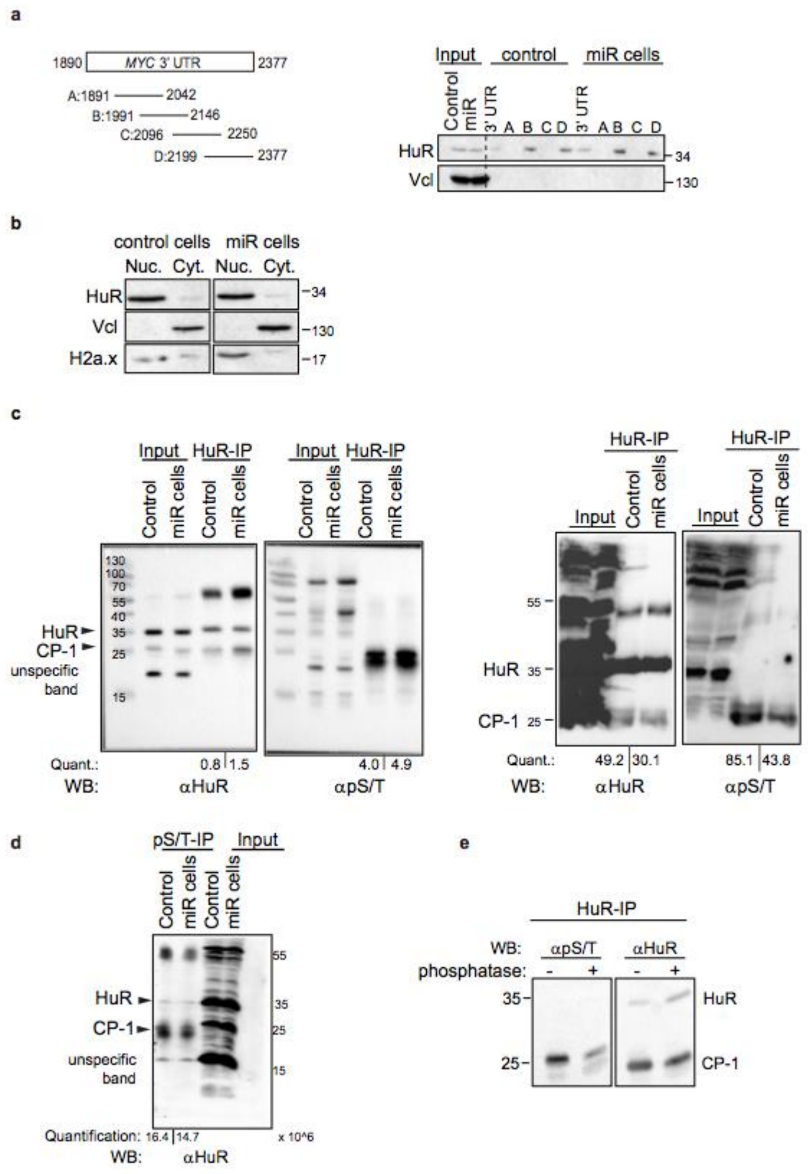

Supplementary Figure 6, related to Figure 4. A mild increase in miR-17-19b level affects HuR phosphorylation and binding to MYC mRNA. (a) Schematic representation of biotinylated RNAs corresponding to four overlapping fragments (A-D) of MYC 3' UTR (left panel), used in RNA pull-down experiment (right panel). The RNA pull-down assay was done with extracts from control and miR cells. HuR and Vcl (used as negative control) were detected by western blot. **(b)** Western blot analysis showed no changes in the subcellular localisation of HuR upon miR-17-19b overexpression. Vcl and H2A.X were used as markers for cytoplasmic and nuclear extracts, respectively. **(c)** Cytoplasmic extracts from control and miR-17-19b overexpressing cells were subjected to IPs using anti-HuR, followed by anti-Phospho-Serine/Threonine (pS/T) western blot analysis. Two experimental replicas are shown. (**d**) Cytoplasmic extracts from control and miR cells were subjected to IP using anti-pS/T antibody, followed by anti-HuR western blot analysis. (**e**) HuR-IPs - treated and untreated with λ-protein phosphatase- were subjected to western blot analysis using anti-HuR and anti-pS/T antibodies.

Mihailovich M. et al., 2015 Supplementary Fig 7

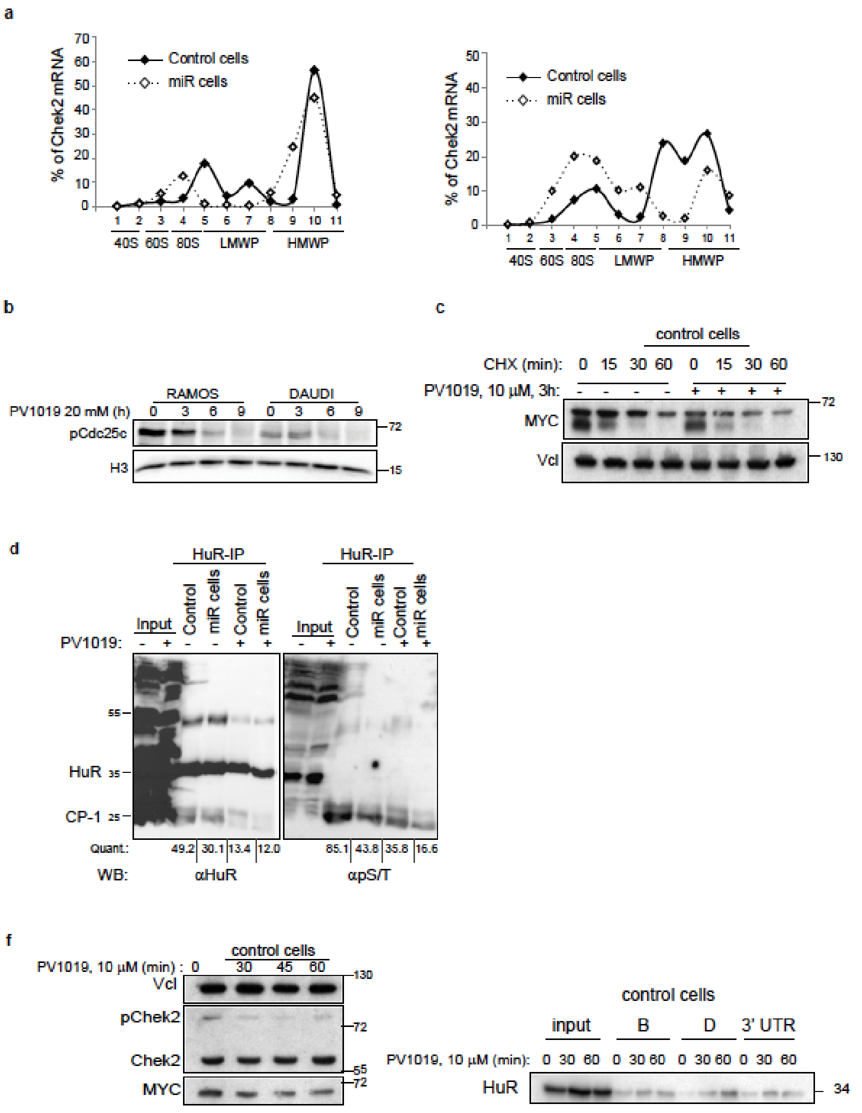

Supplementary Figure 7, related to Figure 5. Chek2 activity regulates MYC expression. (a) Chek2 mRNA translation was assessed using the same polysome gradients as for the *MYC* mRNA (displayed in Supplementary Fig. 5b), starting with equal amounts of cytoplasmic lysates (left panel) or equal starting number of control and miR cells (right panel). 40S and 60S = small and large ribosomal subunits, respectively; 80S = monosomes; LMWP (fractions 6-8) and HMWP (fractions 9-11) = low- and high-molecular weight polysomes, respectively. **(b)** The effectiveness of Chek2 inhibitor, PV1019, was assessed by western blot analysis of Chek2 direct target, pCdc25c, in human BL cell lines, RAMOS and DAUDI. **(c)** Stability of MYC protein is not affected by PV1019 as shown by cycloheximide treatment of PV1019-treated control cells; Vcl was used as loading control. **(d)** Western blot analysis of HuR-IP from miR and control cells (sample obtained within the same experiment diplayed in Supplementary Fig. 6c, right panel) treated with PV1019. **(f)** MYC is down-regulated upon 30 min of PV1019 treatment. Western blot analysis of Chek2, pChek2 and MYC in control cells treated with PV1019 for 0, 30, 45 and 60 min. Vcl was used as a loading control (left panel). HuR binds more efficiently to fragments B and D of *MYC* 3' UTR upon PV1019 treatment in RNA pull-down experiments (right panel).

Mihailovich M. et al., 2015 Supplementary Fig 8

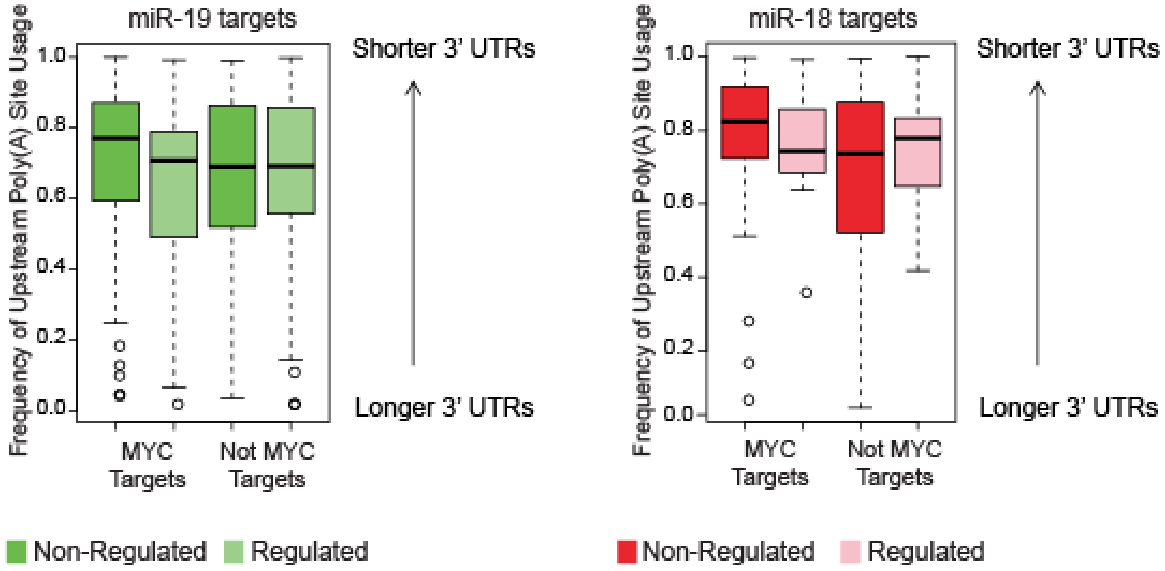

Supplementary Figure 8, related to Figure 7. Frequency of poly(A) site usage analysis for Regulated and Non-regulated miR-19 and miR-18 targets. The comparison between Regulated and Non-regulated targets reveals longer 3' UTRs for the class of regulated targets, but only when they are co-regulated by MYC, for miR-19 targets. The same difference in 3' UTR lengths was not observed for miR-18 targets.

Supplementary Figure 9

Full western blots:

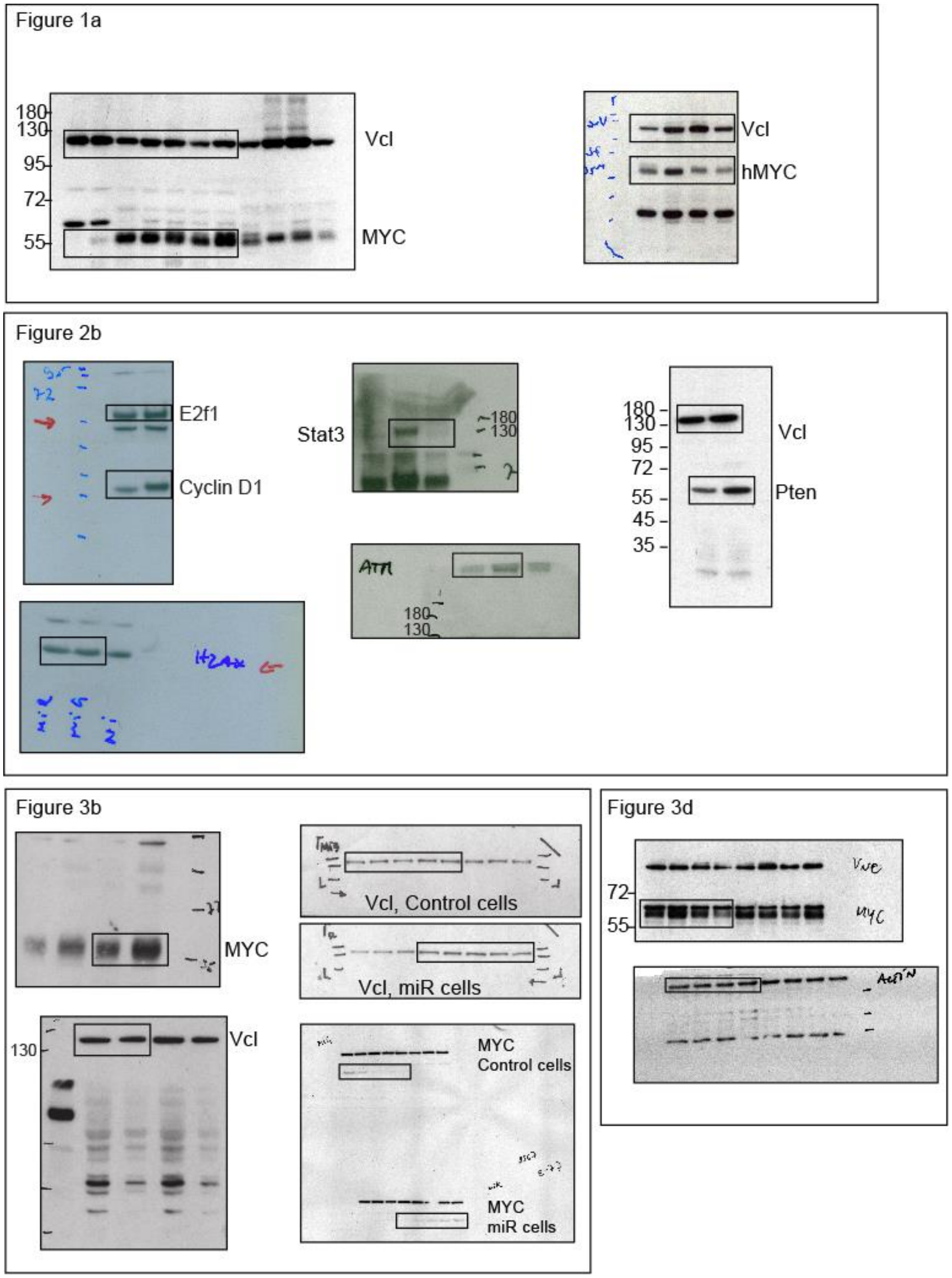

Figure 4

Figure 4a

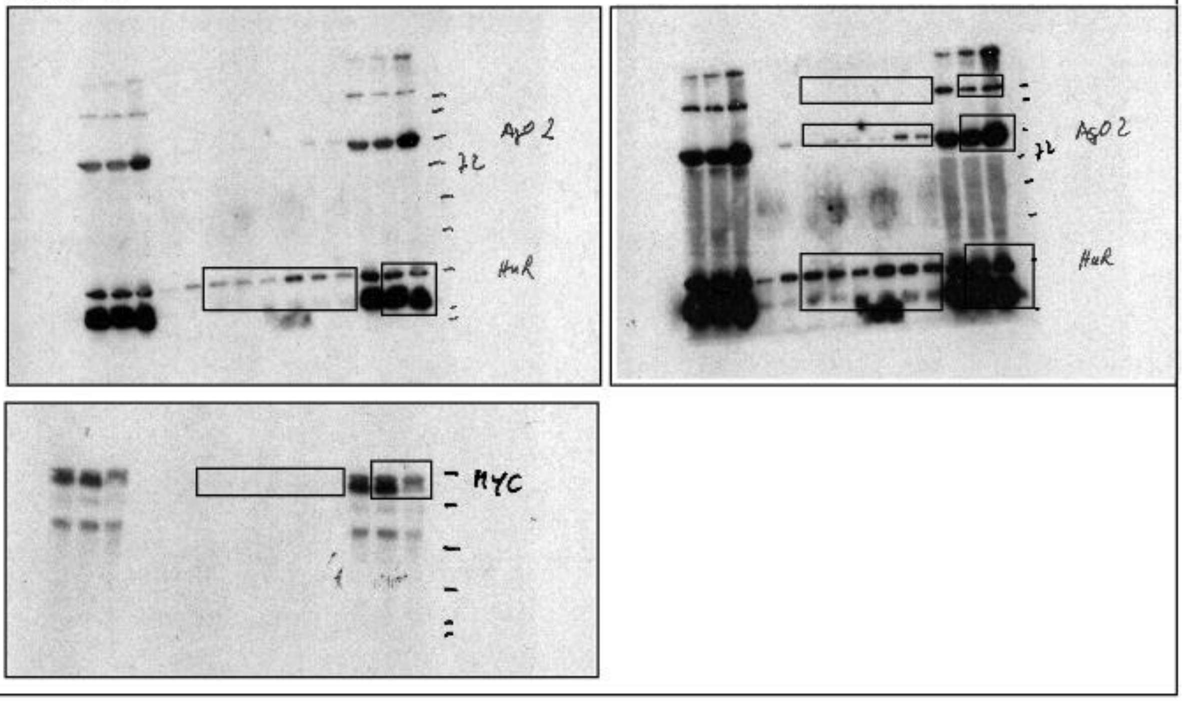

Figure 4b

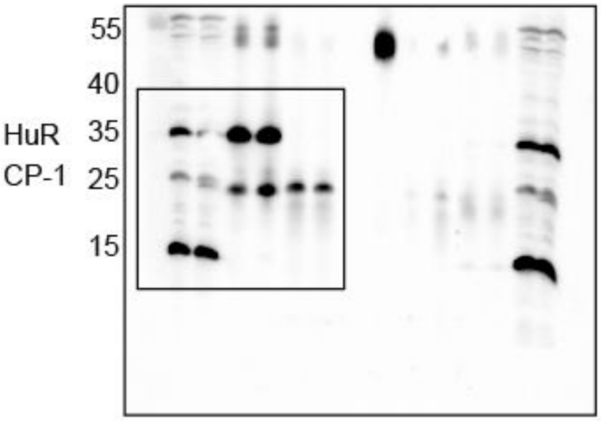

Figure 5

Figure 5a

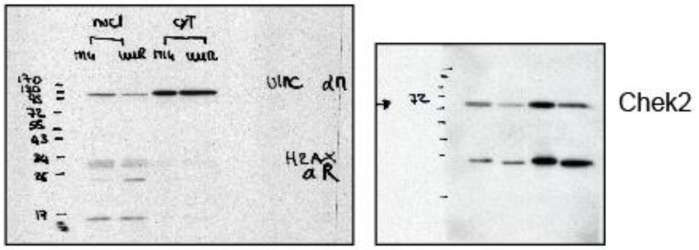

Figure 5d

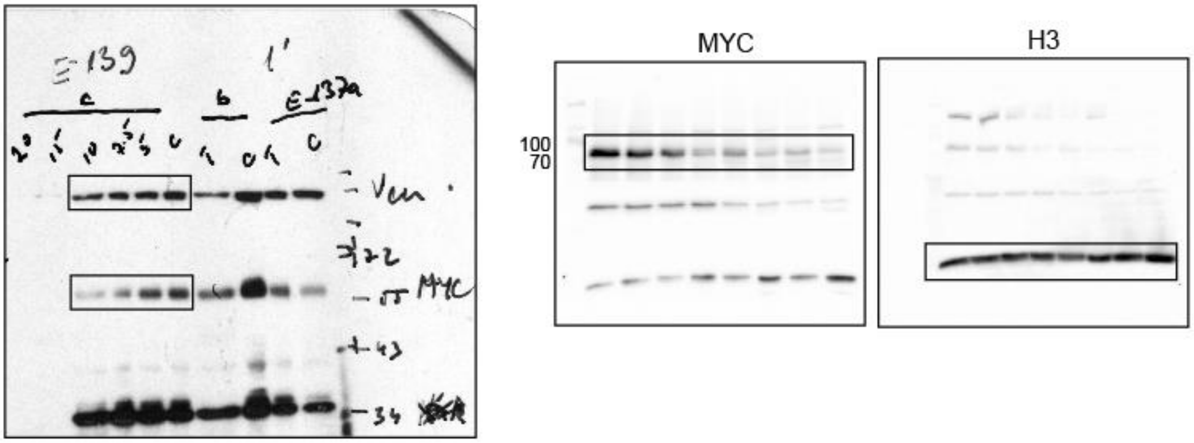

Figure 5e

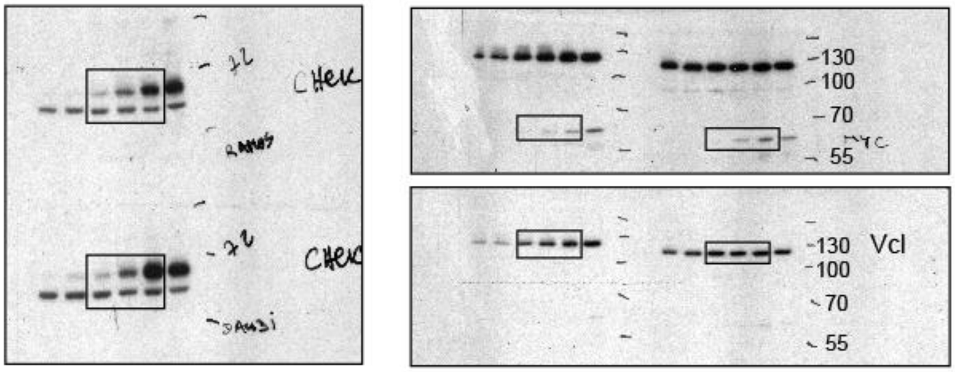

Figure 5f

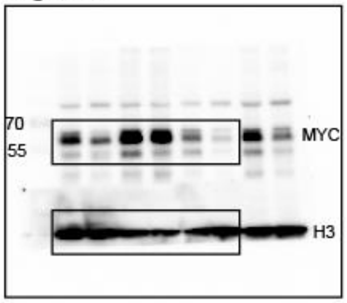

Supplementary Figure 2

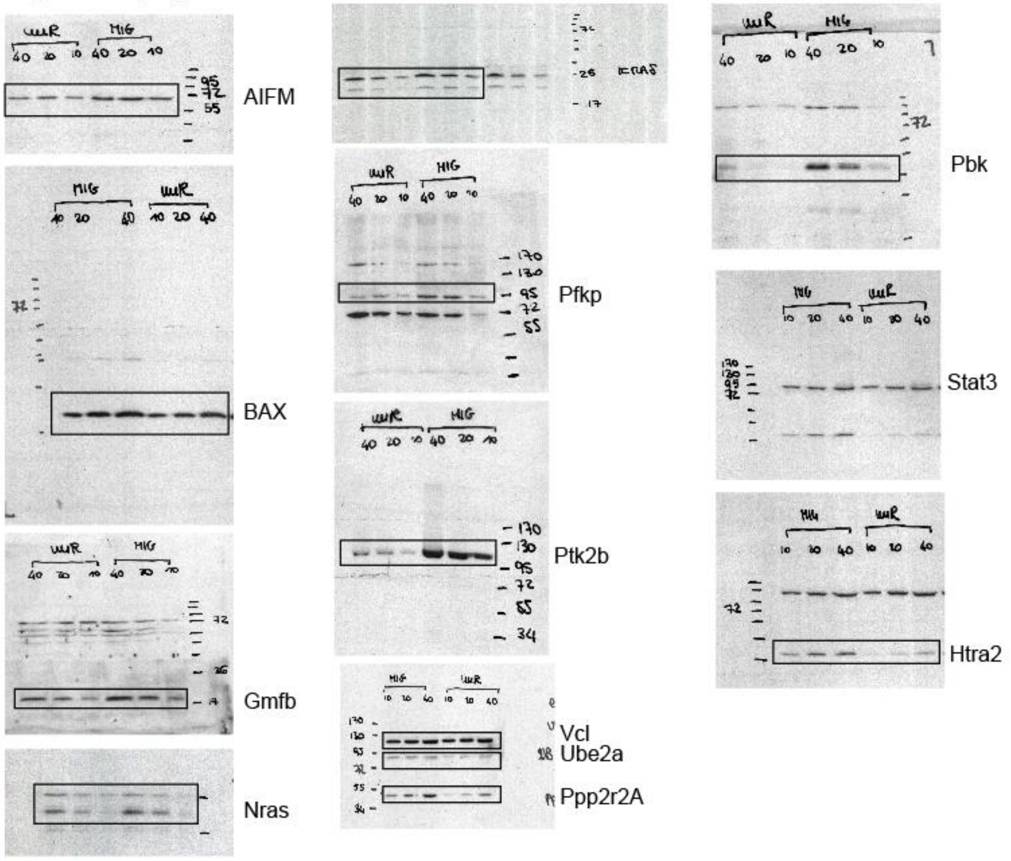

Supplementary Figure 5c

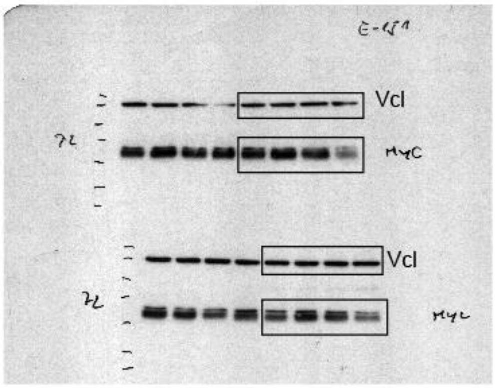

Supplementary Figure 6

Supplementary Figure 6a

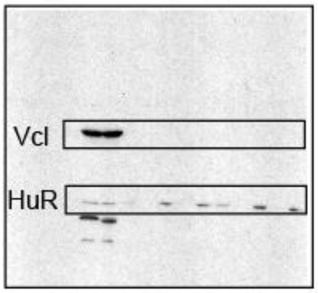

Supplementary Figure 6b

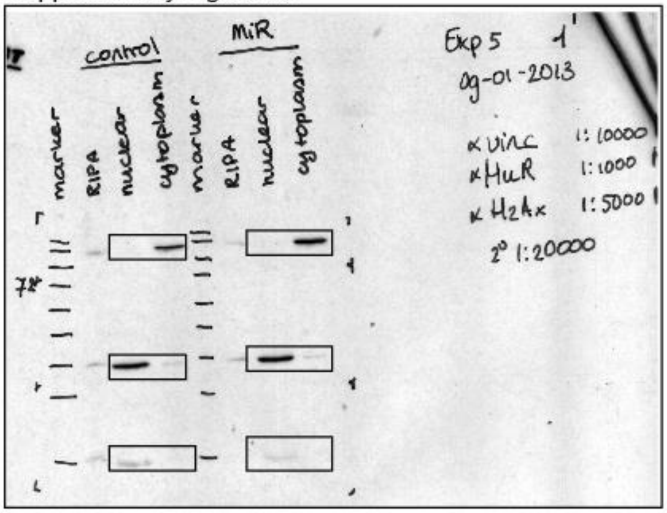

Supplementary Figure 6c

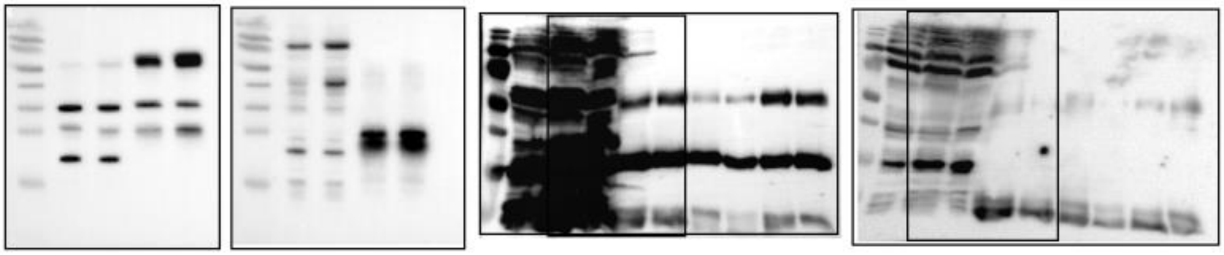

Supplementary Figure 6d

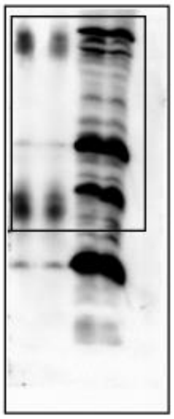

Supplementary Figure 6e

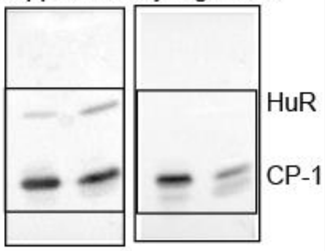

Supplementary Figure 7

Supplementary Figure 7b

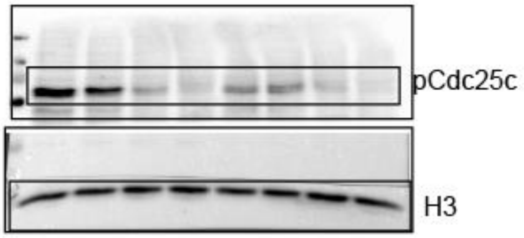

Supplementary Figure 7c

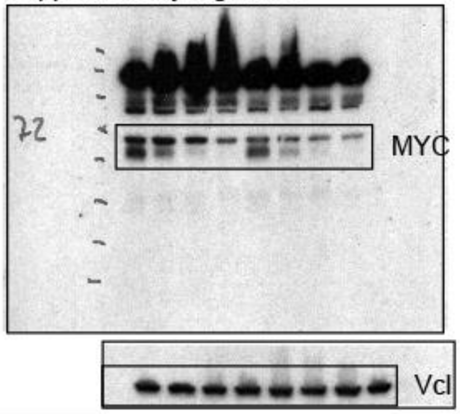

Supplementary Figure 7d

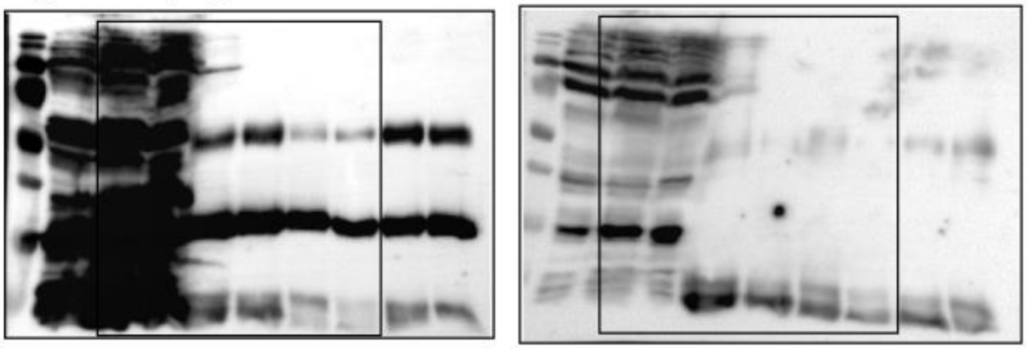

Supplementary Figure 7f

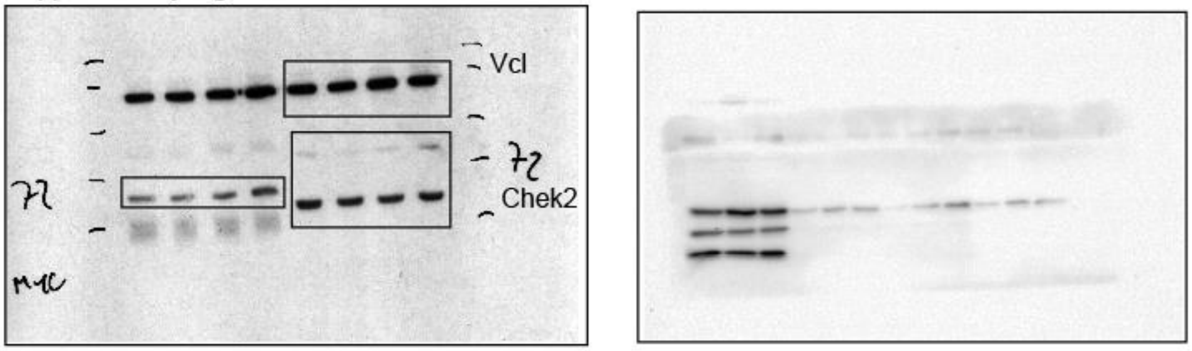

Supplement: Supplementary Information — Supplementary Figures 1-9 [file ncomms9725-s1.pdf]
